# A Systematic Review of the Facilitators and Challenges as Perceived by Dental Service Providers in the Provision of Oral Healthcare for Culturally and Linguistically Diverse Populations

**DOI:** 10.1111/cdoe.70021

**Published:** 2025-09-03

**Authors:** Sudheer Babu Balla, Nikolaos Angelakopoulos, Jyothi Tadakamadla, Santosh Kumar Tadakamadla

**Affiliations:** ^1^ Dentistry and Oral Health La Trobe Rural Health School Bendigo Australia; ^2^ Department of Orthodontics & Dentofacial Orthopaedics University of Bern Bern Switzerland

**Keywords:** CALD, challenges, dental service providers, mixed method, oral healthcare, systematic review

## Abstract

**Objectives:**

Vulnerable groups, particularly those from Culturally and Linguistically Diverse (CALD) backgrounds, face heightened risks of poor oral health. There is a notable gap in systematically analysing the facilitators and challenges dental service providers face in meeting the needs of patients from CALD backgrounds. This study, therefore, sought to systematically review existing literature to address this gap and provide insights into the factors that influence dental service provision for CALD communities.

**Methods:**

A comprehensive search of six electronic databases was conducted to identify facilitators and challenges in oral healthcare provision. Database searches covered January 1985 to May 2025 (last updated 31 May 2025). A meta‐integration approach was employed to synthesise qualitative and quantitative findings. The quality of evidence was assessed using the Mixed Method Appraisal Tool (MMAT). Data organisation followed Ferlie and Shortell's healthcare model, with key themes identified via thematic analysis.

**Results:**

Thirteen papers were included in this review, comprising three quantitative and 10 qualitative studies, sourced from diverse countries, including the United Kingdom, Australia, New Zealand, Japan, Sweden, Netherlands, Finland, Germany, the United States and Canada. Article quality varied from moderate to high. Although individual‐level factors such as cultural beliefs and language barriers, impacted dental service provision and rigid organisational structures also served as a significant challenge. Structural/system‐level challenges included policy implementation gaps, insufficient cross‐cultural training for dental providers and affordability issues.

**Conclusion:**

The evidence from dental service providers in diverse settings suggests they encounter several challenges when providing dental care to CALD communities. In summary, delivering culturally sensitive oral healthcare is inherently complex. Policymakers must acknowledge that addressing the needs of CALD patients necessitates establishing supportive environments and strengthening institutional capabilities.

## 1 Background

According to the World Migration Report, as of 2020, there were an estimated 281 million international migrants worldwide, constituting approximately 3.6% of the global population [1]. International migrants are individuals who reside outside their country of birth for at least 12 months. The majority, about 65% (182 million), were found in high‐income countries, while 31% (86 million) resided in middle‐income nations, predominantly upper‐middle‐income. Comparatively, low‐income countries hosted a smaller proportion of migrants, approximately 4% (13 million) [2]. This trend highlights the increasing prominence of high‐income countries as primary destinations for international migrants, accommodating approximately 75% of migrants between 2000 and 2020 [2]. Migrants can differ significantly in terms of their skill levels, with some entering through highly skilled migration programs, while others migrate as low or unskilled workers to meet labour demands in the host country.

The evolving landscape of cross‐border migration requires a reassessment of health policies to protect the well‐being of the increasingly diverse migrant population effectively [3]. The migration experience significantly influences both physical and mental health, exposing migrants, including refugees, to vulnerabilities such as unemployment, social isolation, racism, discrimination, substandard living conditions and limited access to health services [4]. To enhance the health and well‐being of this population, it is crucial to strengthen health systems, ensuring they are sensitive and inclusive to migrants and refugees. The 2022 World Health Organisation (WHO) report highlighted a global shortage of comprehensive research on health, migration and displacement [5]. Recognising the importance of oral health, the WHO incorporated an oral healthcare resolution into its 2021 political agenda for universal health coverage, aiming to enhance global oral health by 2030 [6]. This resolution emphasises the imperative for actionable steps to strengthen national and international oral health policy agendas by reinforcing health system capabilities.

Migration stands as a significant social determinant of health, including oral health [7]. Migrant groups are often referred to in literature as a Culturally and Linguistically Diverse (CALD) population. The definition of CALD found in reports and policy documents can vary depending on the state and organisation [8]. For instance, according to the Australian Institute of Health and Welfare, CALD pertains to a parent who was born in another country and speaks a language other than English [9]. On the other hand, Dental Health Services Victoria defines CALD as someone who differs from the predominant English‐speaking population, taking into account differences in religion, spirituality, racial backgrounds, ethnicity and language [10]. Despite the absence of a universally agreed‐upon definition, identifying who falls under the category of CALD is adaptable and, therefore, complex. This complexity means that individuals born overseas in English‐speaking countries like Ireland or the United States can also be included in this definition [8]. It is essential to recognise that terminology in this domain varies across international contexts. For instance, the term BAME (Black, Asian and Minority Ethnic) is predominantly employed in the United Kingdom (UK), whereas CALD is more commonly utilised within the Australian context. These terms reflect regional‐specific frameworks and sociopolitical priorities; however, each presents inherent limitations with respect to the accuracy of data collection, inclusivity and the comparability of findings across national boundaries [11]. For the purposes of this review, the designation CALD pertains to individuals who were born outside the host country and primarily communicate in a language other than the official language(s) of that country.

Maintaining oral health for those with a migration background poses a notable challenge due to its intricate connection with various social determinants. These factors extend beyond migrant characteristics, encompassing the contextual environment of destination countries, including the distinctive health systems and cultural values [12, 13]. The provision of oral healthcare exhibits considerable global variation. In many high‐income countries, publicly funded dental services are typically limited in scope and accessibility, often restricted to particular populations, including CALD population subgroups. Conversely, low‐ and middle‐income countries frequently contend with systemic challenges such as workforce shortages, high reliance on out‐of‐pocket payments and limited integration of oral health within broader health systems [5, 6]. Many studies have highlighted a link between migration background and lower oral health‐related quality of life [14, 15, 16, 17, 18, 19]. Although existing literature extensively covers oral health status, perceptions, and access barriers for migrants, there is a distinct gap in research regarding dental service provider (DSP) perspectives [20, 21, 22]. Despite their wealth of experience in healthcare, healthcare providers in different countries often encounter difficulties like language barriers, cultural disparities, and addressing the trauma and mental health needs of CALD patients when striving to deliver optimal care to them [23, 24]. This indicates the necessity to investigate their views and experiences, specifically focusing on the challenges they face when providing dental care. This study, therefore, aimed to systematically review the literature on the facilitators and challenges experienced by DSPs. This systematic review was conducted to address the question: What are the facilitators and challenges for DSPs in addressing the oral health needs of CALD populations?

## 2 Methods

A mixed‐method systematic review design was employed. In public health, stories (qualitative research) have the power to change policies, and statistics (quantitative research) traditionally provide a strong rationale for making changes [25, 26, 27]. Quantitative studies, including cross‐sectional or cohort studies, explore factors influencing DSPs' communication with migrant patients, while qualitative studies, involving interviews or focus group discussions, provide in‐depth insights into the ‘why’ behind these factors. This review was designed, conducted and reported according to the Preferred Reporting Items for Systematic Reviews and Meta‐Analyses (PRISMA) guidelines [28]. It has been registered in the International Prospective Register of Systematic Reviews (PROSPERO) under the registration number #CRD42023482431.

### 2.1 Conceptual Framework

The framework for this review was based on Ferlie and Shortell's Four‐Level Model of Health Care System [29]. According to this model, successful healthcare depends on the integration of the health system across various levels: individual patients, group/team (doctors, supporting staff), organisation (e.g., hospitals or clinics) and larger system/environment (legal, cultural and economic factors). It acknowledges that challenges for healthcare providers stem not only from their attitudes towards migrant patients but also from broader constraints in their working environment. In this review, we organised the gathered data on facilitators and challenges into three components, adapting Ferlie and Shortell's healthcare model [28]. For this review, the findings were categorised under three headings: (1) individual (interaction between DSPs and migrants), (2) organisational (interaction between DSPs and their workplace) and (3) structural/system (related to local, regional and national health care systems, particularly laws and regulations governing the right to healthcare for migrant patients) [30].

### 2.2 Identification of Relevant Literature

This review aimed to provide a comprehensive overview of the challenges and opportunities that DSPs encountered in delivering oral health services to migrant or refugee populations. This review uses the term ‘DSP’ as a general term, encompassing dentists, dental therapists, dental hygienists, oral health therapists, dental auxiliaries (such as dental chairside assistants and dental nurses), and any other healthcare workers involved in providing dental care. On the other hand, people from culturally and linguistically diverse backgrounds were referred to as CALD groups in this review.

### 2.3 Search Strategy

A thorough search was conducted across six electronic databases, MEDLINE, EMBASE, CINAHL, SCOPUS, WEB OF SCIENCE and PROQUEST, utilising both MeSH terms and keywords. The search strategy involved three main concepts. The first focused on keywords specific to CALD populations. The second incorporated terms related to DSPs, encompassing ‘dentists’, ‘dental service provider’, and ‘oral health practitioner’, as well as other professionals such as ‘dental hygienists’, to ensure comprehensive coverage. The third concept included provider‐related keywords like ‘barriers’, ‘facilitators’, ‘experiences’, ‘challenges’, ‘difficulties’, etc. Keywords within each concept were connected with ‘OR’, and the results of each concept were combined using ‘AND’ to obtain a comprehensive result encompassing all three search concepts. Table 1 indicates the search terms employed in the Medline database.

**TABLE 1:** Search terms used in Medline data for the systematic review.

1. “CALD”.mp. 2. “Cultural* and linguistic*”.mp. 3. “non‐English speak*”.mp. 4. “foreign‐born”.mp. 5. “refugee*”.mp. 6. “asylum seeker*”.mp. 7. “migrant*”.mp. 8. “Emigrants”.mp. or “Emigrants and Immigrants”/ 9. “foreign‐background”.mp. 10. 1 or 2 or 3 or 4 or 5 or 6 or 7 or 8 or 9 11. “dentist*”.mp. 12. “dental service provider*”.mp. 13. “dental expert*”.mp. 14. “dental practi*”.mp. 15. “oral health practi*”.mp. 16. “stakeholder*”.mp. 17. 11 or 12 or 13 or 14 or 15 or 16 18. 10 and 17 19. “experience*”.mp. 20. “challenge*”.mp. 21. “barrier*”.mp. 22. “difficult*”.mp. 23. “perception*”.mp. 24. “perspective*”.mp. 25. “facilitator*”.mp. 26. 19 or 20 or 21 or 22 or 23 or 24 or 25 27. 18 and 26 28. “mixed‐method*”.mp. 29. “qualitative study”.mp. 30. “quantitative study”.mp. 31. 28 or 29 or 30 32. 27 and 31

### 2.4 Study Selection & Eligibility Criteria

Two reviewers (SBB & NA) independently screened all the titles and abstracts within the initial selection. Any discrepancies were resolved through discussion until a consensus was achieved. The subsequent phase involved a comprehensive review of the articles that passed the initial screening, where the reviewers analysed the key messages. Any disagreements at this stage were resolved through discussion with a third reviewer (JT). Eligible studies met the following criteria: (1) providing information on perceptions OR attitudes OR barriers OR facilitators OR challenges for DSPs implementing oral health care for CALD populations, (2) being peer‐reviewed studies (qualitative, quantitative, or mixed‐methods) with information relevant to CALD populations and (3) constituting primary research. Articles were excluded if they met any of the following criteria: (1) DSPs' perceptions and attitudes not relevant to the study's topic, (2) studies exploring DSPs' experiences providing care to population groups other than CALD, such as indigenous people or domestic migrants (individuals who have relocated within the same country), (3) study protocols, opinion pieces, policy papers and guidelines and (4) publication in languages other than English. Any uncertainties regarding article inclusion were deliberated among all authors using the specified eligibility criteria before a final decision was reached.

### 2.5 Quality Appraisal

Studies meeting the inclusion criteria were assessed using the Mixed Method Appraisal Tool (MMAT, 2018) [31]. It assesses the methodological rigour of diverse study designs, encompassing qualitative research, randomised controlled trials, non‐randomised studies, quantitative descriptive studies and mixed‐methods research. There are two screening questions at the beginning of the tool, which are common for all study designs. These questions focus on aspects like the clarity of the research question and whether the collected data addressed it. Following the screening questions are the appraisal questions, which differ according to the study design. Each appraisal question was rated as ‘yes’, ‘no’, or ‘can't tell’. The tool results in a methodological rating of < 1 or 1 (being the highest quality) based on the evaluation of study selection bias, study design, data collection methods, sample size and analysis [30]. The first reviewer (SBB) conducted an initial quality assessment for each paper, encompassing both qualitative and quantitative aspects. The findings were then compared with the independent appraisal of the second reviewer (NA). Any discrepancies between the reviewers were resolved through discussion. No articles were excluded based on the quality assessment score, as all were considered significant for this review [32, 33].

### 2.6 Data Extraction and Synthesis

**FIGURE 1:**
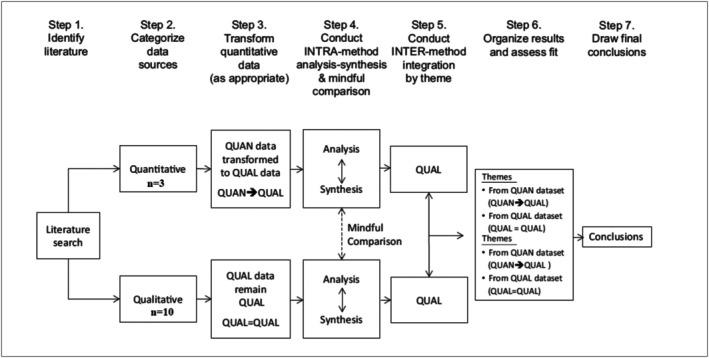
Convergent qualitative meta integration. *Source:* Adapted from Frantzen and Fetters [34].

The analytical approach leveraged meta‐integration of the basic convergent qualitative synthesis type. Within this meta‐integration, the qualitative data remain as the qualitative data (QUAL = QUAL), and quantitative data are transformed into qualitative data (QUAN → QUAL) (Figure 1) [34]. Results from all included studies (both quantitative and qualitative) were transformed into qualitative findings to present relevant codes and themes [26]. Utilising NVivo 14 for Windows software (QSR International Pty Ltd) [35], the qualitative dataset was independently analysed by two reviewers (SBB and NA). Thematic analysis was conducted using a systematic six‐step process: data familiarisation, initial code generation, theme identification, theme review, theme definition and naming and report generation [36]. Each text line was carefully read, interpreted and coded based on content and contextual meaning. Similar codes were grouped, and a comparative analysis was conducted between the two reviewers (SBB and NA) to discern similarities and disparities. These codes were subsequently amalgamated into hierarchical themes, wherein initial codes were subsumed into broader categories. The final overarching themes were collaboratively agreed upon by both reviewers (SBB and NA) and validated by the third and fourth authors (JT and SKT). The extracted data were then systematically mapped onto Ferlie and Shortell's healthcare system model [29], further delineated into facilitators and challenges for oral healthcare provision to CALD populations across three levels: individual, organisational and structural/system.

## 3 Results

**FIGURE 2:**
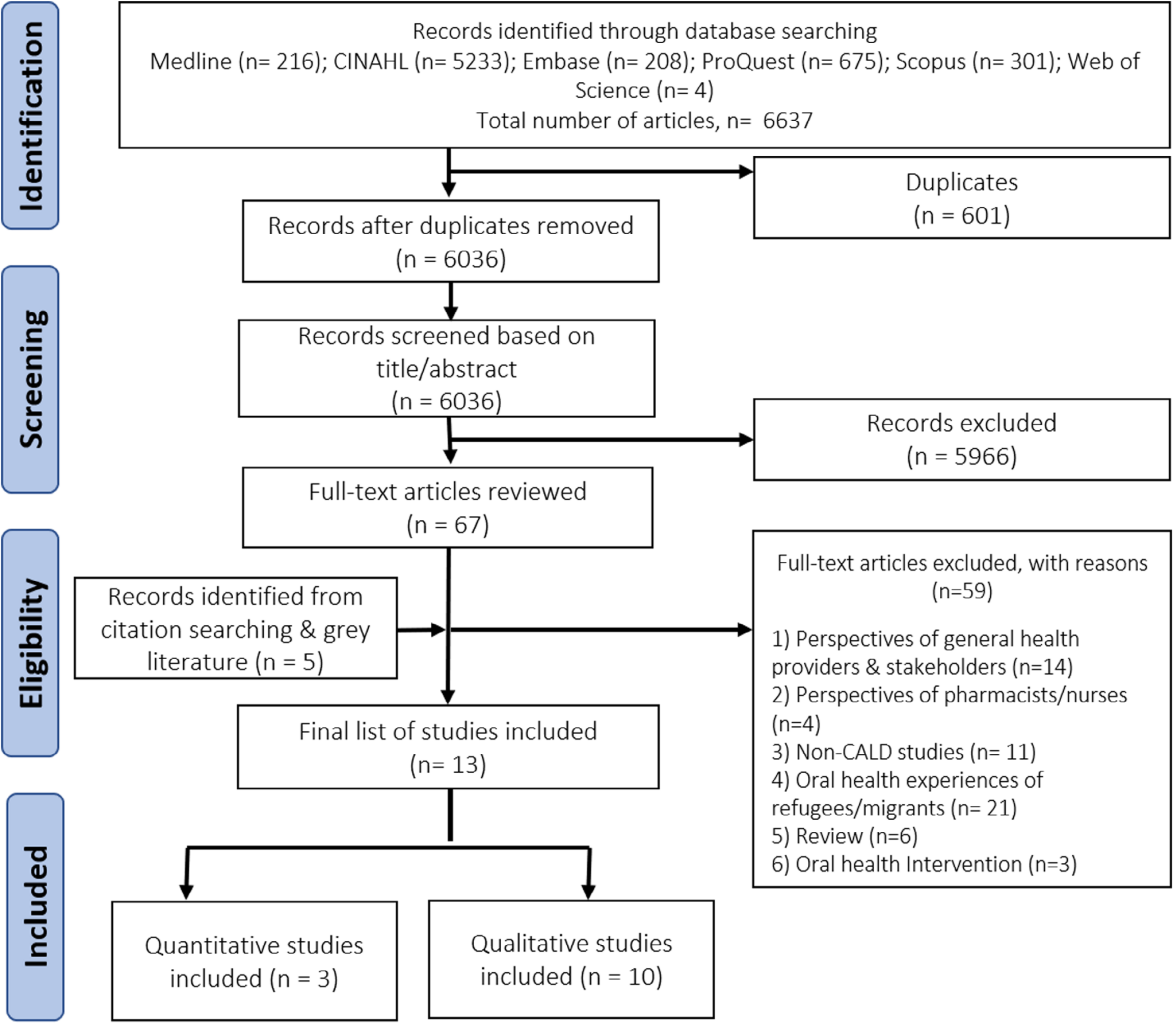
PRISMA flow chart of study selection.

Of 6637 studies retrieved from searching the databases, 6036 remained after removing duplicates and foreign language articles for title and abstract screening. Subsequently, 67 full‐text articles were evaluated, with 13 meeting the inclusion criteria. Articles were excluded due to the focus on non‐CALD populations, reviews, barriers and facilitators experienced by the CALD population, or general practitioner‐centred topics (Figure 2).

### 3.1 Study Characteristics

The review consisted of 13 articles, which were conducted and published between 1985 and May 2025. Among the 13 included studies, the majority were conducted in the United Kingdom (*n* = 3) [37, 38, 39], followed by Australia (*n* = 2) [40, 41]. There was one study each from Sweden [42], New Zealand [43], Netherlands [44], Japan [45], Finland [46], Germany [47], United States of America [48] and Canada [49]. Ten studies utilised qualitative data collection methods [38, 39, 41, 43, 44, 45, 46, 47, 48, 49], while three employed quantitative approaches [37, 40, 42]. Most studies (*n* = 9) involved interviews with dentists, oral health practitioners, or community dental officers [37, 38, 40, 41, 42, 43, 44, 45, 46], with one study focusing on experts in dental migrant research and dental practitioners [47] and two studies focusing on dental hygienists and dental hygiene students [48, 49]. Another study engaged various stakeholders within dental settings, including administration, community engagement, policy and dental practitioners [39]. Overall, the total number of participants across all studies was 1065, varying between 4 and 593 (Table 2).

**TABLE 2:** Study characteristics.

Author/year of publication	Location/country	Type and design of the study	Recruitment, data collection	Participant population, sample size	Population served	Outcomes	Data quality score
Widstrom, 1985 [42]	Stockholm/Sweden	Quantitative, descriptive cross‐sectional	Private practicing dentists and Public Dental Services in the 10 local council areas with a high percentage of immigrants. Questionnaire	176 private practicing dentists and 34 public dental services	Turkish, Spanish, Arabic, and Finnish patients	Language difficulties, poor knowledge of dentistry, difficulties in keeping treatment times, and exaggerated expression of pain as frequent problems	1
Williams et al., 1995 [37]	Leeds/United Kingdom	Quantitative, descriptive cross‐sectional	Community & general dental practitioners working in locations with a high proportion of resident Asian populations were recruited. Questionnaire	593 general dental practitioners and 46 community dental officers	Asian populations	Language barrier is the major impediment to care, patient's understanding of the treatment proposed, gaining consent, and difficulty obtaining medical histories	0.8
Goldsmith et al., 2005 [40]	Western Australia	Quantitative, descriptive cross‐sectional	The Western Australian branch of the Australian Dental Association Newsletter. Questionnaire	120 (77% were general dental practitioners and 23% were specialists)	Italian, Chinese, and Vietnamese	Language‐related communication barriers and compromised informed consent	0.6
Zhang, 2008 [43]	Wellington/New Zealand	Qualitative descriptive	Dental clinics in the Wellington area Semi‐structured interviews	4 (Two private dental surgeons and two community‐based dental providers)	Chinese	Cost of dental care, language problems, a lack of knowledge of dental health, a low priority given to oral health care, mixed attitudes towards dentists, lack of information, making appointments, and transportation impediments	0.6
Aljafari et al., 2015 [38]	London/England	Qualitative descriptive	General dental practitioners working in the referral area for King's College Hospital Semi‐structured interviews	18 general dental practitioners	Information is not provided	Child's young age, poor cooperation, and high treatment need, parental attitudes, social inequality, exclusion and cultural barriers, and inadequate secondary care communication and engagement	1
Due et al., 2020 [41]	Adelaide/Australia	Qualitative descriptive	From oral health clinics with a known history of working with refugees or asylum seekers Semi‐structured interviews	6 oral health practitioners	Middle‐eastern refugees and asylum seekers	Delayed oral health care, cultural norms, negative consequences of resettlement, and prior bad dental care experiences	1
Van Midde et al., 2020 [44]	Amsterdam/Netherlands	Qualitative descriptive	Dentists within the voluntary dental network Semi‐structured interviews	7 dentists	Information is not provided	Lack of personal connection or gratitude from the patient, financial limitations, urgent oral health needs, and compassion and trust	1
Imafuku et al., 2022 [45]	Gifu/Japan	Qualitative descriptive	Recruited dentists from mid‐size regional cities in Japan Semi‐structured interviews	11 dentists	Asian populations	Linguistic, sociolinguistic, and sociocultural	1
Paajanen et al., 2022 [46]	Helsinki, Finland	Qualitative descriptive	Dentists from Helsinki municipality public dental care Semi‐structured interviews	6 dentists	Information is not provided	Language barrier, cultural differences, and prejudice	1
Paisi et al., 2022 [39]	Plymouth, United Kingdom	Qualitative descriptive	From a dental setting (details not mentioned) Online semi‐structured interviews	12 stakeholders working with ASRs. Five of them working within a dental setting that included dentists	Refugees and Asylum‐Seekers	Prioritising survival and safety, cultural norms and practices, lack of dental care knowledge, financial limitations, and accessibility issues	1
Spinler et al., 2022 [47]	Hamburg, Germany	Qualitative descriptive	Practicing dentists and representatives of German dental board Semi‐structured interviews	9 expert interviews	Turkish migrants	Language, perceived significance of oral health, oral health knowledge, health socialisation, and patient‐dentist interaction	1
Capozzi et al., 2018 [48]	Boston, USA	Qualitative descriptive	Senior dental hygiene students (DHS) from the Massachusetts College of Pharmacy and Health Sciences Bachelor of Science program Semi‐structured interviews	18 dental hygiene students	Information is not provided	—	1
Charbonneau et al., 2014 [49]	British Columbia, Canada	Qualitative descriptive	Recruited from a private dental office Focus group	5 dental hygienists	Information is not provided	Cultural competence (CC), the difficulties of practising CC, education needs related to CC, and CC in oral health care education	1

In the three quantitative studies, questionnaires were distributed to dental practitioners and community dental officers. One study used Likert‐type questions [40], another utilised open‐ended questions grouped by themes [42], and the third employed multiple‐choice questions [37]. The 10 included qualitative studies centred on DSPs' experiences and challenges in providing oral healthcare to CALD populations, except for one focusing on providers' perspectives regarding voluntary dental care for undocumented migrants [44].

### 3.2 Quality Assessment

The quality assessment revealed a high risk of nonresponse bias in two quantitative studies [37, 40]. Regarding qualitative studies, all articles meticulously detailed the research questions, methodologies and rationale behind the chosen data collection methods. One qualitative paper published in 1985 provided insufficient information on most appraisal parameters [43]. The inter‐reviewer reliability for quality assessment for qualitative and quantitative studies was 90% and 92%, respectively. Discrepancies in the reliability scores were later resolved through discussion between the reviewers. Table S1 summarises individual study quality assessments.

### 3.3 Thematic Analysis

**FIGURE 3:**
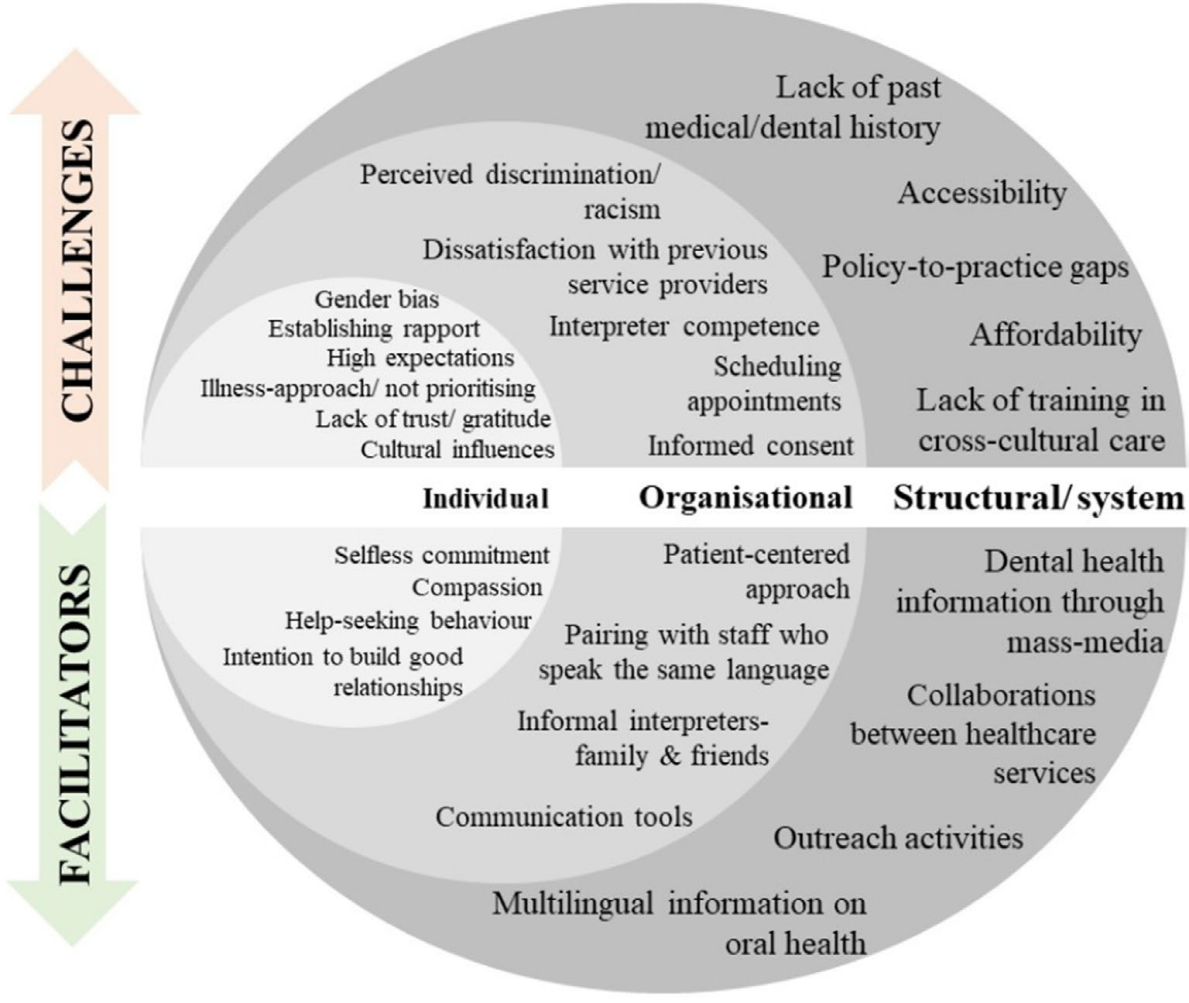
Facilitators and challenges of dental service providers in the provision of oral healthcare for CALD population groups fitted into Ferlie and Shortell's healthcare model [29].

After conducting thematic analysis and synthesis, five overarching analytical themes emerged regarding the facilitators and challenges encountered by DSPs when delivering oral healthcare to the CALD population. Figure 3 illustrates that the facilitators and challenges associated with the provision of oral healthcare to CALD populations, as identified from the included studies, span all levels of Ferlie and Shortell's healthcare model [29], ranging from the individual to the system level. Table S2 presents the themes and subthemes identified from the data analysis with example quotes.

#### 3.3.1 Theme 1: Cultural Factors

Theme 1 encompasses the knowledge, attitudes, beliefs and cultural influences of CALD patients regarding oral healthcare, which could act as facilitators and challenges for DSPs in providing oral healthcare services. This theme comprises four descriptive subthemes: oral health literacy of CALD patients, awareness and understanding of available dental services, cultural beliefs related to accessing dental services, and beliefs and attitudes towards oral hygiene practices. DSPs identified limited oral health knowledge and awareness of preventive measures among migrants as a significant challenge [38, 43, 47]. One study identified patients' lack of awareness regarding where to seek dental care and whom to contact, indicating gaps in accessing dental services [39]. Additionally, DSPs noted challenges for CALD patients, such as failing to establish regular dental attendance patterns and not seeking preventive care, influenced by language barriers and socio‐cultural factors affecting patients' understanding of dental care [38]. Cultural perceptions among CALD patients were also identified as challenges to oral healthcare provision. For instance, Chinese migrants perceived dental caries as caused by air inside their bodies rather than bacteria [43]. Moreover, the taboo surrounding the expression of pain among CALD patients was noted, with expressing pain being considered a sign of weakness [39]. The attitudes of CALD individuals towards oral health care were perceived as challenges for oral healthcare provision by DSPs [41]. Furthermore, gender bias in patient‐DSP interactions was also posed as a challenge by DSPs. Paajanen et al.'s [46] study highlights situations where male patients doubt the female DSPs' professionalism and knowledge, as well as scenarios where patients may express preference or discomfort based on the dentist's gender, particularly in cases where a male dentist is expected to treat a female patient.

#### 3.3.2 Theme 2: Language/Communication Factors

The theme of ‘Language/Communication factors’ in providing oral healthcare to CALD patients emerges prominently in the discourse surrounding the facilitators and challenges encountered by DSPs. This theme encompasses various subthemes, including establishing rapport, gathering past medical/dental history, methods of communication, conveying treatment information, obtaining informed consent and communication facilitated by interpreters.

DSPs highlighted the difficulty in establishing rapport with migrant patients, considering it a significant obstacle to delivering oral healthcare [38]. Some providers resorted to referring patients to other practitioners due to communication barriers [40]. Additionally, some providers perceived communicating with migrant patients as more demanding and energy‐intensive [46]. Confidence in treating CALD patients among DSPs was notably higher when comprehensive information about patients' symptoms and medical/dental history was provided [37]. However, many reported challenges in obtaining this crucial information, describing it as cumbersome [40, 47]. Although some providers found traditional communication aids such as hand gestures or dictionaries ineffective, others advocated for alternative methods such as diagrams, models, or diagnostic imaging to improve communication [40]. Few providers suggested adapting communication styles to better align with their patients' cultural expectations and needs [45].

DSPs also highlighted the challenges they encounter in effectively communicating the long‐term implications of treatment plans to migrant patients, especially in scenarios where language barriers are prevalent [37, 40, 42, 45]. Conversely, some providers emphasised the importance of leveraging modern dental practice management software, incorporating visual treatment planning features, or providing oral care information in multiple languages to improve patients' understanding of complex procedures and treatment options [40, 45, 48]. Moreover, concerns were raised regarding compromised informed consent due to language barriers, as some providers observed reduced opportunities for CALD patients to ask questions and actively engage in treatment decisions [37, 40].

DSPs provided insights into their perspectives on utilising interpreter services in delivering oral healthcare to CALD patients. Although some studies highlighted the significant challenge posed by the unavailability of interpreters, leading to the denial of dental care access [39], others expressed concerns regarding the varying proficiency levels among interpreters, potential distractions affecting communication [40, 42, 46], and the benefits of professional interpreters in offering clear explanations and facilitating informed consent [40, 46]. In addition to these, discussions also encompassed the use of untrained or informal interpreters, with DSPs expressing reservations regarding translation accuracy, comprehension of complex oral healthcare concepts, privacy and legal implications [40, 45]. However, it was acknowledged that informal interpreters are preferred for their accessibility, promptness and cost‐effectiveness, often seen as fostering trust between DSPs and CALD patients [40].

#### 3.3.3 Theme 3: Psychosocial Factors

The challenges encountered by DSPs in delivering oral healthcare to migrant patients revolved around psychosocial factors. These encompassed various subthemes, including the lack of gratitude, frustration and fear experienced by DSPs, ethical dilemmas in treatment decision‐making, disparities between patient expectations and reality, influences of previous dental experiences, effects of resettlement or traumatic pasts, providers' selfless commitment and compassion towards CALD patients, and the attitudes of CALD patients towards seeking help.

DSPs frequently reported feelings of disappointment when CALD patients expressed dissatisfaction with care, despite receiving professional care. In a study of Chinese migrants in New Zealand, some patients distrusted necessary treatments, such as extractions for periodontal reasons, due to limited oral health literacy and differing cultural beliefs, further contributing to providers' frustration [43]. Similarly, when undocumented migrants received free treatment through a voluntary network, dissatisfaction often arose from unmet expectations for cosmetic or complex procedures, leading to frustration among providers who had invested significant time and effort [44]. Additionally, some providers reported apprehension, fear and frustration when encountering challenges in obtaining patient consent and when interpretation services were unavailable [39]. Ethical dilemmas arose for providers when determining treatment options as they struggled to balance the needs of CALD patients, impacting patient experiences and outcomes [44]. Practical constraints were also noted due to unexpected complications during treatment [44]. Some providers described how CALD patients' preferences resulted in incomplete treatment and missed preventive care opportunities [45]. In contrast, others discussed how ineffective communication influenced treatment decision‐making, leading to a sense of unfinished treatment and dissatisfaction with care quality [45].

In one study from Japan, explaining the intricacies of the Japanese health insurance system to migrant patients, especially concerning their preferences for specific dental treatments, emerged as a challenge for DSPs, highlighting the complexity of managing CALD patient expectations [45]. Additionally, providers discussed the difficulty of meeting CALD patients' expectations while ensuring treatment solutions align, emphasising how misunderstandings can lead to patients perceiving discrimination [46]. Negative experiences, such as errors in dental treatment or providers' reluctance to accept patients from CALD backgrounds, were identified as factors that may undermine patients' trust and confidence in dental care providers [39, 41]. DSPs also highlighted the challenges encountered during the resettlement period of humanitarian migrants, recognising the influence of past traumatic experiences, unstable living conditions and unresolved asylum seeker status on patients' prioritisation of oral health [39, 41, 46]. Conversely, the selfless commitment, sensitivity, cultural awareness, open‐mindedness, non‐judgmental attitude and compassion exhibited by DSPs, along with their proactive help‐seeking attitude, were identified as facilitators in providing oral healthcare to CALD populations [38, 39, 40, 44, 45, 46, 48].

#### 3.3.4 Theme 4: Affordability

This theme incorporates two main subthemes: cost and resource limitations. Although only one study viewed the cost of dental care as a significant challenge to oral healthcare provision [43], another study elucidated how out‐of‐pocket payment obligations for dental treatments contribute to avoidance behaviours and altering treatment decisions, thereby hindering DSPs in delivering optimal oral healthcare [44, 47]. DSPs also addressed how financial limitations affect dietary choices among CALD populations, leading to adverse oral health outcomes and necessitating extensive dental treatment [39].

#### 3.3.5 Theme 5: Structural and System Determinants

DSPs identify structural and systemic determinants as the third most commonly cited challenge to delivering oral healthcare to CALD populations. This theme encompasses various subthemes, including dentist training, clinic burdens/losses, oral health education, system support, access to services, policy‐to‐practice gaps and the importance of key partnerships and meaningful relationships. DSPs outlined the hurdles in communication and coordination within healthcare systems. A UK‐based study involving the providers underscored the challenges within the National Health Services (NHS) in effectively liaising with local authorities and organisations aiding vulnerable groups. Additionally, they shed light on the access disparities and waiting list hurdles within the NHS, perceived to prioritise the general populace over vulnerable groups [39]. Some providers noted that accessing dental services for CALD populations is notably challenging, particularly in urgent appointment scenarios, highlighting this as a potential barrier to oral healthcare provision [39].

Several DSPs emphasised the insufficient training offered in dental schools as a barrier to adequately treating patients with limited English proficiency [40]. Conversely, a few dentists raised concerns about the financial implications of allocating staff resources to interpretation services, expressing worries about the strain on practice efficiency. They also noted potential challenges in maintaining scheduling efficiency and accommodating a larger patient volume, which could impact the practice's financial sustainability [40]. On the contrary, DSPs highlighted several facilitators to oral healthcare provision, including adapting educational materials to diverse cultural backgrounds and linguistic preferences [37, 39], disseminating oral health information through mass media and immigrant associations [42], conducting proactive outreach activities to educate and empower migrant communities [42], implementing positive incentive schemes for dentists [36] and enhancing awareness of service locations and transportation options [39]. Few providers recommended lectures for DSPs, offering them insights on specific cultures [47]. A policy‐to‐practice gap was identified, as reported by Riggs et al. [50], where both community members and maternal healthcare staff in Victoria, Australia, were unaware of government policies providing free dental care for pregnant refugee women, despite existing provisions and referral pathways. They also emphasised the significance of improving oral health accessibility through collaborative partnerships and integration, matching providers with CALD patients who share the same language and fostering meaningful relationships through a patient‐centric approach to care delivery and service design [37, 39].

## 4 Discussion

This systematic review, employing a mixed‐method approach, addressed the question: ‘What are the facilitators and challenges for DSPs in addressing oral health needs of CALD patients?’ Understanding the perspectives of dental professionals regarding oral healthcare for CALD individuals is essential for promoting health equity, enhancing health outcomes, and ensuring equitable access to high‐quality dental services. This review amalgamated data from numerous studies spanning diverse countries and cultural contexts, spanning over four decades, and involving more than a thousand DSPs. Through systematic analysis and critical assessment, 13 studies encompassing various qualitative and quantitative designs were evaluated, elucidating the facilitators and challenges encountered by DSPs in catering to the oral health needs of CALD patients.

This systematic review identified various facilitators and challenges affecting the delivery of comprehensive oral healthcare to CALD communities, including cultural factors, language/communication factors, psychosocial factors, affordability and structural/system determinants. The analysis elucidates a complex interplay of factors across individual, organisational and structural/system levels within the healthcare model proposed by Ferlie and Shortell [29]. At the individual level, sociocultural differences in oral health norms, practices and beliefs between CALD populations' countries of origin and the host country can influence their understanding and utilisation of dental care, contributing to service provision challenges. Organisational challenges arise from communication barriers, leading to compromised informed consent, which is influenced by interpreter proficiency in dental terminology. Structural/system factors, including difficulties in access, funding gaps and inadequate provider training, further hinder the provision of oral healthcare to CALD populations.

This integrated mixed‐method systematic review revealed an intricate interconnection among the individual, organisational and structural/system levels, where each aspect influences the others. For example, the oral health knowledge of CALD patients at the individual level may impact their ability to access dental services at the structural level [51]. This underscores the importance of implementing culturally sensitive communication strategies and outreach programs on a broader scale to enhance oral healthcare accessibility for CALD patients. Likewise, cultural influences of CALD patients on oral health at the individual level could shape the treatment decisions made by DSPs at the organisational level. For instance, the cultural values held by CALD patients, like only seeking dental treatment when experiencing severe pain, could pose a significant challenge for DSPs [52]. The provision of services for CALD populations is continually evolving and can be affected by various factors. These include the attitudes of healthcare providers, the specific needs of CALD populations, factors within the healthcare system, legal framework and societal values [30]. The interaction and complexity among these factors can either hinder or improve the delivery of comprehensive oral healthcare to CALD patients.

### 4.1 Individual Level

The facilitators and challenges identified at this level highlight the influence of CALD patients' knowledge, attitudes and cultural beliefs on oral healthcare provision by DSPs. As societal dynamics evolve, understanding diverse behaviours becomes imperative, especially in the provider‐patient relationship, which can be challenging yet pivotal in healthcare delivery [53]. Given CALD patients' retention of native languages and traditions, healthcare providers must enhance their cultural competence [49, 54]. In this review, compassionate care stood out in person‐centred care, demonstrated by DSPs' empathy, commitment and efforts to foster meaningful relationships. These include adjusting communication to accommodate linguistic diversity, adopting patient‐centred approaches and promoting help‐seeking behaviours, all of which facilitate oral healthcare provision at the individual level.

Cultural beliefs among CALD patients were perceived by DSPs as barriers to effective oral healthcare provision; for example, among some Chinese migrants, dental caries were attributed to the presence of ‘air’ in the body rather than bacterial infection [43], while in other cultural contexts, the expression of pain was perceived as a sign of weakness, leading to delay in seeking dental care [39]. Furthermore, cultural beliefs, particularly those related to gender preferences, were found to significantly complicate oral healthcare provision. One study included in this review reported that DSPs encountered challenges stemming from gender bias and perceptions held by CALD patients in clinical settings [46]. These findings align with previous research indicating that female healthcare professionals often felt disrespected by certain CALD patients [55, 56]. This dynamic instilled a fear among service providers of being accused of racism if they unintentionally made cultural missteps in their clinical practice [57, 58]. Additionally, the issue of mistrust was compounded by CALD patients' past negative experiences with DSPs, their expectations shaped by previous encounters, or their unfamiliarity with the healthcare system [59, 60]. Puranik and Kumar suggested that patient preferences regarding the gender of the DSP vary depending on assumed general qualities attributed to the genders such as favouring female DSPs for their empathetic approach and male DSPs for their professional expertise. However, it is essential to note that this study did not involve CALD participants, limiting its relevance to this population [61].

### 4.2 Organisational Level

While individual‐level factors significantly influence patient‐provider interactions, these challenges are further compounded by systemic issues within healthcare organisations. Delivering culturally sensitive healthcare is paramount for enhancing the oral health outcomes of CALD patients. However, translating this concept into practical application poses challenges, as evidenced in a recent review [30]. For instance, utilising interpreters presents a notable dilemma. In nations where English is predominantly spoken, CALD people often face obstacles due to limited English proficiency, impeding their ability to engage effectively with the healthcare system. Consequently, this leads to restricted access and exacerbated health disparities [62]. Although several studies acknowledge the value of employing interpreting services, they also highlight the associated drawbacks such as the required time and effort. This is particularly pertinent given the financial and human resources constraints within healthcare settings [39, 40, 42, 45, 46]. Merely employing bilingual interpreters does not automatically ensure the delivery of high‐quality, culturally sensitive care, as research findings indicate. DSPs' concerns include translation accuracy and the interpreters' familiarity with dental terminology. Furthermore, challenges arise when interpreters lack insight into migrants' cultural behaviours and beliefs despite bridging the language gap [63, 64].

Despite the presence of language service policies, standards and guidelines mandating the utilisation of interpreter services, there is a noticeable tendency in several countries, including Australia, to underutilise them. Instead, there is a preference for ad hoc interpreters such as family members, friends and untrained individuals [65]. This trend raises concerns among DSPs regarding the accuracy of translation, privacy issues and legal implications. However, some providers view ad hoc interpreters favourably due to their convenience, accessibility, speed, cost‐effectiveness and ability to foster patient trust. Addressing language barriers requires a multifaceted approach beyond simply recruiting interpreters. It entails tailoring services to meet the specific needs of CALD patients effectively.

Alongside the importance of high‐quality interpretation, the absence of ethnic diversity among healthcare staff emerged as another significant challenge in delivering cross‐cultural oral healthcare. Williams et al. [37] investigated the DSP's concerns about providing care for British Asians in the UK. They underscored the necessity of recruiting Asian dental staff to act as cultural intermediaries, mainly as many were unaware of Asian cultural nuances. Furthermore, respondents in the study by Paisi et al. [39] highlighted the potential significance of pairing CALD patients with dentists who speak the same language to mitigate any cultural barriers effectively.

Delivering oral healthcare in a culturally sensitive manner without CALD patients feeling discriminated against or unfairly treated due to their race is a significant challenge. In one study by Paajanen et al., DSPs highlighted how cultural misunderstandings could lead to patients perceiving discrimination in their healthcare experiences. For example, they noted that treatments like removable dentures, which are standard in some cultures, might be misunderstood by CALD patients as being offered solely because of their immigrant status [46]. In another scenario, DSPs faced difficulties explaining Japan's health insurance system to foreign patients. This highlights the struggle healthcare providers encounter in conveying insurance coverage limitations, potentially leading to CALD patients feeling discriminated against [45]. Worth et al. [58] referred to this challenge as the ‘fear of making cultural blunders’ among health practitioners. Although racism might not be explicitly expressed in oral health practice, Zhang's [43] study observed a negative attitude from one dentist who referred to Chinese migrants as patients not worth his time, indicating reluctance towards them.

Ensuring adequate resources, including allocating more time for providers, is essential in cross‐cultural care. Some providers in this review stressed the importance of flexibility in scheduling appointments and dedicating enough time during consultations to cater to the intricate healthcare requirements of CALD patients [39]. Nadeau and Measham also underscored the need to allocate additional time and resources to effectively address the needs of CALD patients and the significance of collaborating with other services to ensure a comprehensive healthcare approach [66].

### 4.3 Structural/System Level

Access to dental care is crucial for CALD communities, particularly refugees, who have endured trauma that has impacted their oral health [67]. Government policies in many countries and states prioritise certain groups, including pregnant women, children, refugees and asylum seekers, for free public dental care, exempting them from general waitlists. For instance, most states in Australia provide free care to priority populations [68]. However, translating these policies into practice faces significant challenges, revealing notable gaps. For example, in qualitative research undertaken by Riggs et al. in the state of Victoria in Australia, it was found that neither community members nor maternal healthcare staff were aware of this policy. Despite maternity and dental services being available under one umbrella and provisions for midwives to refer pregnant refugee women with poor oral health for dental care, the midwives lacked awareness of referral pathways and government policies [50]. This review identified a significant finding regarding the lack of effective communication channels among various organisations. Paisi et al., in their research focusing on DSPs in the UK, underscored the insufficient communication between the NHS, local authorities and groups supporting vulnerable populations. This results in a lack of awareness regarding how to access dental services [39]. Additionally, the review highlights the necessity of integrating oral health education and dental referral mechanisms into healthcare pathways for better oral healthcare provision.

Recognising and understanding diverse cultures was considered essential for delivering quality care. Some providers assessed that the training they received in dental school for managing patients with limited English skills was inadequate [39]. However, some DSPs proposed the introduction of courses during university education to enhance their understanding of the cultures of the people they work with [40, 48, 49]. Priebe et al. [69] and Khatri and Assefa [70] recommended including cultural training in the education of future service providers to ensure they offer culturally appropriate care. Another significant finding highlighted the challenge of accessing medical or dental history for migrant patients. Participants mentioned that this information is crucial for making accurate diagnoses and effective treatment plans with low risks. Priebe et al. [69] also noted concerns among service providers about not knowing patients' allergies, vaccination history, or past health issues, which could complicate treatment decisions.

In addition to training for DSPs, another crucial aspect of good practice involves disseminating information on oral health and facilitating access to dental services for CALD communities through educational programs and materials. It is acknowledged that there exists a scarcity of information available in multiple languages, and a considerable portion of the population is unaware of the significance of regular healthcare [39]. Priebe et al. [69] highlighted the importance of creating information materials that are easy and inexpensive to produce and finding effective ways to distribute them to CALD communities.

### 4.4 Strengths, Weaknesses and Limitations

To the best of our knowledge, this study represents the first attempt to compile evidence concerning the facilitators and challenges perceived by DSPs in delivering oral healthcare to patients from CALD backgrounds. The systematic review adhered to the methodological and reporting standards outlined by PRISMA [28], and a meta‐integration approach was employed for analysis, following a basic convergent qualitative synthesis method [34]. This integrated approach and thematic analysis allowed for a structured identification of key themes and sub‐themes across various study designs. The mixed‐method design ensured comprehensive data collection from qualitative and quantitative research findings on oral healthcare provision to CALD communities. The quality of evidence was assessed using the MMAT tool, indicating that most studies are of moderate to high methodological quality [31].

However, this study is subject to several limitations. All included studies originated from high‐income countries, which may have constrained the diversity and scope of the evidence base. It is important to note that access to oral healthcare for CALD populations varies significantly across countries, influenced by national policies and regulatory frameworks. For example, in Victoria, Australia, dental health policies prioritise access for refugees and asylum seekers [68], whereas in Germany, medical treatment vouchers are provided, albeit with restrictions on dental care access [71]. These disparities highlight the context‐specific nature of the findings and caution against their generalisation beyond these settings. Furthermore, the perceptions and challenges reported by DSPs were primarily based on subjective self‐reports, without independent verification of actual clinical practices. Additionally, inconsistencies in the use of CALD‐related terminology across studies, where some employed ethnic‐specific labels rather than broader descriptors, may have resulted in the omission of relevant literature during the search process. At last, the potential for publication bias must be acknowledged, as studies reporting significant or positive findings are more likely to be published and indexed in major databases.

## 5 Conclusion

The integrated, mixed‐method systematic review findings highlight the challenges and opportunities experienced by DSPs in developed countries in the provision of oral healthcare for CALD communities. The challenges identified were not linked to particular migrant origins but were universally acknowledged. Factors such as diverse cultural beliefs of patients, language disparities, organisational inflexibility, fear of being perceived as discriminating among DSPs, and interpreter‐related issues impacted service provision at individual and organisational levels. Structural/system‐level challenges included policy to practice gaps, insufficient cross‐cultural training for DSPs, and affordability issues resulting in the accumulation of treatment needs in CALD people. Strategies such as enhancing interpretation services, providing multilingual materials, offering cultural competency training for staff, and recruiting DSPs from similar ethnic backgrounds are recommended to mitigate these challenges and enhance oral healthcare for CALD communities. For example, it is evident from the literature that small interventions, such as incorporating interpreters and limited English proficiency patient training into medical curricula, significantly improved students' cross‐cultural communication skills and confidence in working with diverse populations [72]. This research will aid in developing appropriate care systems that align with the oral health needs of migrants and meet the expectations of DSPs.

## Author Contributions

S.B.B., J.T. and S.K.T. were involved in conceptualising the systematic review. S.B.B. conducted the search strategy across multiple databases, while S.B.B. and N.A. conducted the quality appraisal of studies and data synthesis. J.T. and S.K.T. oversaw the review process and verified the data. S.B.B. drafted the manuscript, and all authors reviewed and approved the final version.

## Ethics Statement

The authors have nothing to report.

## Consent

The authors have nothing to report.

## Conflicts of Interest

The authors declare no conflicts of interest.

## Supporting information


**Table S1:** Individual study quality assessments.
**Table S2:** Themes and subthemes identified from the data analysis with example quotes.
**Table S3:** Search strategies from various data sources.
**Table S4:** Table of excluded studies.

## Data Availability

The data that supports the findings of this study are available in Supporting Information of this article.

## References

[1] International Organisation for Migration, United Nations , “World Migration Report 2022,” (2021).

[2] United Nations Department of Economic and Social Affairs, Population Division , “International Migration 2020 Highlights,” (2020).

[3] D. W. Macpherson , B. D. Gushulak , and L. Macdonald , “Health and Foreign Policy: Influences of Migration and Population Mobility,” Bulletin of the World Health Organization 85, no. 3 (2007): 200–206. 17486211 10.2471/BLT.06.036962 PMC2636223

[4] World Health Organization , “Refugee and Migrant Health,” accessed March 15, 2024, https://www.who.int/health‐topics/refugee‐and‐migrant‐health#tab=tab_1.

[5] World Health Organization , World Report on the Health of Refugees and Migrants (World Health Organization, 2022).

[6] WHO , “Oral Health: Achieving Better Oral Health as Part of the Universal Health Coverage and Noncommunicable Disease Agendas Towards 2030,” Contract No.: EB148/8, (2020).

[7] Y. Sano and T. Abada , “Immigration as a Social Determinant of Oral Health: Does the ‘Healthy Immigrant Effect’ Extend to Self‐Rated Oral Health in Ontario, Canada?,” Canadian Ethnic Studies 51, no. 1 (2019): 135–156.

[8] K. Marcus , M. Balasubramanian , S. Short , and W. Sohn , “Culturally and Linguistically Diverse (CALD): Terminology and Standards in Reducing Healthcare Inequalities,” Australian and New Zealand Journal of Public Health 46, no. 1 (2022): 7–9. 34902191 10.1111/1753-6405.13190

[9] Australian Institute of Health and Welfare , Australia's Health 2018: Culturally and Linguistically Diverse Populations (AIHW, 2018).

[10] Vic Department of Human Services , Culturally and Linguistically Diverse Communities Resource Kit (State Government of Victoria, 2010).

[11] N. Ahmed , “Improving Access to Oral Health and Dental Services for BAME Communities,” General Dental Council, (2020), https://www.gdc‐uk.org/news‐blogs/blog/detail/blogs/2020/09/08/improving‐access‐to‐oral‐health‐and‐dental‐services‐for‐bame‐communities.

[12] D. Malmusi , C. Borrell , and J. Benach , “Migration‐Related Health Inequalities: Showing the Complex Interactions Between Gender, Social Class and Place of Origin,” Social Science & Medicine 71, no. 9 (2010): 1610–1619. 20869798 10.1016/j.socscimed.2010.07.043

[13] J. Spallek , H. Zeeb , and O. Razum , “What Do We Have to Know From Migrants' Past Exposures to Understand Their Health Status? A Life Course Approach,” Emerging Themes in Epidemiology 8, no. 1 (2011): 6. 21843354 10.1186/1742-7622-8-6 PMC3169503

[14] G. Aarabi , C. Walther , B. Kretzler , L. Zwar , H. H. König , and A. Hajek , “Association Between Migration and Oral Health‐Related Quality of Life: Results From a Nationally Representative Online Survey,” BMC Oral Health 22, no. 1 (2022): 309. 35883079 10.1186/s12903-022-02337-5 PMC9321273

[15] N. Xu , S. Deng , Y. Liang , et al., “Impact of Migration on Oral Health Outcomes of Children in Multi‐Beneficial Kindergartens in Nanning, Southern China: A Cross‐Sectional Study,” BMC Oral Health 23, no. 1 (2023): 507. 37480059 10.1186/s12903-023-03212-7 PMC10362724

[16] D. Lauritano , G. Moreo , F. Carinci , V. Campanella , F. Della Vella , and M. Petruzzi , “Oral Health Status Among Migrants From Middle‐ and Low‐Income Countries to Europe: A Systematic Review,” International Journal of Environmental Research and Public Health 18, no. 22 (2021): 12203. 34831957 10.3390/ijerph182212203 PMC8624247

[17] S. Liu , V. Chongsuvivatwong , S. Zhang , and A. Thearmontree , “Effects of Parental Migration on Dental Caries of Six‐ to Eight‐Year‐Old Children Using Structural Equation Modeling,” International Journal of Environmental Research and Public Health 19, no. 20 (2022): 13470. 36294059 10.3390/ijerph192013470 PMC9602841

[18] S. Liu , A. Thearmontree , V. Chongsuvivatwong , S. Zhang , and L. Zhang , “Association Between Parental Migration and Dental Caries of 3‐12‐Year‐Old Children in China: A Systematic Review and Meta‐Analysis,” Journal of International Oral Health 15, no. 5 (2023): 409–417.

[19] R. Qiu , Y. Li , M. Malla , et al., “Impact of Parental Migration on Oral Health Outcomes of Left‐Behind School‐Aged Children in Luchuan, Southern China,” BMC Oral Health 18, no. 1 (2018): 207. 30537963 10.1186/s12903-018-0683-3 PMC6290493

[20] E. Riggs , L. Gibbs , N. Kilpatrick , et al., “Breaking Down the Barriers: A Qualitative Study to Understand Child Oral Health in Refugee and Migrant Communities in Australia,” Ethnicity & Health 20, no. 3 (2015): 241–257. 24739019 10.1080/13557858.2014.907391

[21] G. Aarabi , D. R. Reissmann , U. Seedorf , H. Becher , G. Heydecke , and C. Kofahl , “Oral Health and Access to Dental Care—A Comparison of Elderly Migrants and Non‐Migrants in Germany,” Ethnicity & Health 23, no. 7 (2018): 703–717. 28277023 10.1080/13557858.2017.1294658

[22] T. L. Finlayson , N. Y. Beltran , and K. Becerra , “Psychosocial Factors and Oral Health Practices of Preschool‐Aged Children: A Qualitative Study With Hispanic Mothers,” Ethnicity & Health 24, no. 1 (2019): 94–112. 28398070 10.1080/13557858.2017.1315366

[23] S. Hultsjö and K. Hjelm , “Immigrants in Emergency Care: Swedish Health Care Staff's Experiences,” International Nursing Review 52, no. 4 (2005): 276–285. 16238724 10.1111/j.1466-7657.2005.00418.x

[24] S. Abbott and M. Riga , “Delivering Services to the Bangladeshi Community: The Views of Healthcare Professionals in East London,” Public Health 121, no. 12 (2007): 935–941. 17655892 10.1016/j.puhe.2007.04.014

[25] T. B. Newman , “The Power of Stories Over Statistics,” BMJ 327, no. 7429 (2003): 1424–1427. 14684635 10.1136/bmj.327.7429.1424 PMC300791

[26] P. Pluye and Q. N. Hong , “Combining the Power of Stories and the Power of Numbers: Mixed Methods Research and Mixed Studies Reviews,” Annual Review of Public Health 35 (2014): 29–45. 10.1146/annurev-publhealth-032013-182440 24188053

[27] M. Sandelowski , C. I. Voils , and J. Barroso , “Defining and Designing Mixed Research Synthesis Studies,” Research in the Schools 13, no. 1 (2006): 29. 20098638 PMC2809982

[28] M. J. Page , J. E. McKenzie , P. M. Bossuyt , et al., “The PRISMA 2020 Statement: An Updated Guideline for Reporting Systematic Reviews,” BMJ 372 (2021): n71. 33782057 10.1136/bmj.n71 PMC8005924

[29] E. B. Ferlie and S. M. Shortell , “Improving the Quality of Health Care in the United Kingdom and the United States: A Framework for Change,” Milbank Quarterly 79, no. 2 (2001): 281–315. 11439467 10.1111/1468-0009.00206 PMC2751188

[30] R. Suphanchaimat , K. Kantamaturapoj , W. Putthasri , and P. Prakongsai , “Challenges in the Provision of Healthcare Services for Migrants: A Systematic Review Through Providers' Lens,” BMC Health Services Research 15 (2015): 390. 26380969 10.1186/s12913-015-1065-z PMC4574510

[31] Q. N. Hong , S. Fàbregues , G. Bartlett , et al., “The Mixed Methods Appraisal Tool (MMAT) Version 2018 for Information Professionals and Researchers,” Education for Information 34 (2018): 285–291.

[32] M. Dixon‐Woods , S. Bonas , A. Booth , et al., “How Can Systematic Reviews Incorporate Qualitative Research? A Critical Perspective,” Qualitative Research 6, no. 1 (2006): 27–44.

[33] C. Pope , N. Mays , and J. Popay , Synthesizing Qualitative and Quantitative Health Evidence: A Guide to Methods (Open University Press, McGraw Hill Education, 2007).

[34] K. K. Frantzen and M. D. Fetters , “Meta‐Integration for Synthesizing Data in a Systematic Mixed Studies Review: Insights From Research on Autism Spectrum Disorder,” Quality and Quantity 50 (2016): 2251–2277.

[35] QSR International Pty Ltd , “NVivo Qualitative Data Analysis. Version 14 [Software],” (2018), https://support.qsrinternational.com/nvivo/s/.

[36] V. Braun and V. Clarke , “Using Thematic Analysis in Psychology,” Qualitative Research in Psychology 3, no. 2 (2006): 77–101.

[37] S. A. Williams , J. H. Godson , and I. A. Ahmed , “Dentists' Perceptions of Difficulties Encountered in Providing Dental Care for British Asians,” Community Dental Health 12, no. 1 (1995): 30–34. 7697561

[38] A. K. Aljafari , J. E. Gallagher , and M. T. Hosey , “Failure on All Fronts: General Dental Practitioners' Views on Promoting Oral Health in High Caries Risk Children—A Qualitative Study,” BMC Oral Health 15 (2015): 45. 25888427 10.1186/s12903-015-0032-8 PMC4403841

[39] M. Paisi , R. Baines , H. Wheat , et al., “Factors Affecting Oral Health Care for Asylum Seekers and Refugees in England: A Qualitative Study of Key Stakeholders' Perspectives and Experiences,” British Dental Journal 232, no. 11 (2022): 1–7. 35676462 10.1038/s41415-022-4340-5 PMC9176155

[40] C. Goldsmith , L. Slack‐Smith , and G. Davies , “Dentist‐Patient Communication in the Multilingual Dental Setting,” Australian Dental Journal 50, no. 4 (2005): 235–241. 17016888 10.1111/j.1834-7819.2005.tb00366.x

[41] C. Due , I. Aldam , and A. Ziersch , “Understanding Oral Health Help‐Seeking Among Middle Eastern Refugees and Asylum Seekers in Australia: An Exploratory Study,” Community Dentistry and Oral Epidemiology 48, no. 3 (2020): 188–194. 32131149 10.1111/cdoe.12524

[42] E. Widström , “Dentists' Experiences of Immigrants as Patients,” Swedish Dental Journal 9, no. 6 (1985): 243–247. 3868831

[43] W. Zhang , “Oral Health Service Needs and Barriers for Chinese Migrants in the Wellington Area,” New Zealand Dental Journal 104, no. 3 (2008): 78–83. 18980048

[44] M. van Midde , I. Hesse , G. J. van der Heijden , et al., “Access to Oral Health Care for Undocumented Migrants: Perspectives of Actors Involved in a Voluntary Dental Network in The Netherlands,” Community Dentistry and Oral Epidemiology 49, no. 4 (2021): 330–336. 33341949 10.1111/cdoe.12605

[45] R. Imafuku , Y. Nagatani , and M. Shoji , “Communication Management Processes of Dentists Providing Healthcare for Migrants With Limited Japanese Proficiency,” International Journal of Environmental Research and Public Health 19, no. 22 (2022): 14672. 36429391 10.3390/ijerph192214672 PMC9690798

[46] A. Paajanen , T. Karaharju‐Suvanto , J. Koivumäki , and M. Kaila , “Finnish Dentists' Experiences With Foreign‐Background Patients—A Qualitative Study,” Acta Odontologica Scandinavica 80, no. 7 (2022): 529–534. 35276056 10.1080/00016357.2022.2047779

[47] K. Spinler , C. Kofahl , E. Ungoreit , G. Heydecke , D. Dingoyan , and G. Aarabi , “Access Barriers to Dental Treatment and Prevention for Turkish Migrants in Germany ‐ A Qualitative Survey,” Frontiers in Public Health 10 (2022): 862832. 35692338 10.3389/fpubh.2022.862832 PMC9178233

[48] B. M. Capozzi , L. J. Giblin‐Scanlon , and L. Rainchuso , “Treatment of a Culturally Diverse Refugee Population: Dental Hygiene Students' Perceptions and Experiences,” Journal of Dental Hygiene 92, no. 2 (2018): 50–56. 29739847

[49] C. J. Charbonneau , D. M. Kelly , and L. R. Donnelly , “Exploring the Views of and Challenges Experienced by Dental Hygienists Practising in a Multicultural Society: A Pilot Study,” Canadian Journal of Dental Hygiene 48, no. 4 (2014): 139–146.

[50] E. Riggs , J. Yelland , R. Shankumar , and N. Kilpatrick , “‘We Are All Scared for the Baby’: Promoting Access to Dental Services for Refugee Background Women During Pregnancy,” BMC Pregnancy and Childbirth 16 (2016): 12. 26794243 10.1186/s12884-015-0787-6 PMC4722780

[51] K. Divaris , J. Y. Lee , A. D. Baker , and W. F. Vann, Jr. , “Caregivers' Oral Health Literacy and Their Young Children's Oral Health‐Related Quality‐of‐Life,” Acta Odontologica Scandinavica 70, no. 5 (2012): 390–397. 22150574 10.3109/00016357.2011.629627 PMC3305855

[52] R. Mariño , V. Minichiello , and M. I. Macentee , “Understanding Oral Health Beliefs and Practices Among Cantonese‐Speaking Older Australians,” Australasian Journal on Ageing 29, no. 1 (2010): 21–26. 20398082 10.1111/j.1741-6612.2010.00395.x

[53] E. Ng , “The Healthy Immigrant Effect and Mortality Rates,” Health Reports 22, no. 4 (2011): 25–29. 22352149

[54] M. Klenner , R. Mariño , P. Pineda , G. Espinoza , and C. Zaror , “Cultural Competence in the Nursing, Dentistry, and Medicine Professional Curricula: A Qualitative Review,” BMC Medical Education 22, no. 1 (2022): 686. 36127655 10.1186/s12909-022-03743-7 PMC9485016

[55] A. C. Dalheim Englund and I. Rydström , “‘I Have to Turn Myself Inside Out’: Caring for Immigrant Families of Children With Asthma,” Clinical Nursing Research 21, no. 2 (2012): 224–242. 22473272 10.1177/1054773812438915

[56] S. Høye and E. Severinsson , “Intensive Care Nurses' Encounters With Multicultural Families in Norway: An Exploratory Study,” Intensive & Critical Care Nursing 24, no. 6 (2008): 338–348. 18468898 10.1016/j.iccn.2008.03.007

[57] L. Manirankunda , J. Loos , P. Debackaere , and C. Nöstlinger , “‘It Is not Easy’: Challenges for Provider‐Initiated HIV Testing and Counseling in Flanders,” Belgium AIDS Education and Prevention 24, no. 5 (2012): 456–468. 23016506 10.1521/aeap.2012.24.5.456

[58] A. Worth , T. Irshad , R. Bhopal , et al., “Vulnerability and Access to Care for South Asian Sikh and Muslim Patients With Life Limiting Illness in Scotland: Prospective Longitudinal Qualitative Study,” BMJ 338 (2009): b183. 19190015 10.1136/bmj.b183 PMC2636416

[59] J. M. O'Mahony and T. T. Donnelly , “The Influence of Culture on Immigrant Women's Mental Health Care Experiences From the Perspectives of Health Care Providers,” Issues in Mental Health Nursing 28, no. 5 (2007): 453–471. 17613147 10.1080/01612840701344464

[60] S. Sandhu , N. V. Bjerre , M. Dauvrin , et al., “Experiences With Treating Immigrants: A Qualitative Study in Mental Health Services Across 16 European Countries,” Social Psychiatry and Psychiatric Epidemiology 48, no. 1 (2013): 105–116. 22714866 10.1007/s00127-012-0528-3

[61] M. P. Puranik and A. Kumar , “Gender Stereotypes in Dental Care: Across‐Sectional Study,” Journal of Pharmaceutical and Biomedical Sciences 5, no. 12 (2015): 941–945.

[62] M. D. Silva , R. D. Adelman , V. Singh , et al., “Healthcare Provider Perspectives Regarding Use of Medical Interpreters During End‐of‐Life Conversations With Limited English Proficient Patients,” American Journal of Hospice & Palliative Care 39, no. 2 (2022): 220–227. 34000817 10.1177/10499091211015916 PMC10080979

[63] A. Bischoff and P. Hudelson , “Access to Healthcare Interpreter Services: Where Are We and Where Do We Need to Go?,” International Journal of Environmental Research and Public Health 7, no. 7 (2010): 2838–2844. 20717543 10.3390/ijerph7072838 PMC2922730

[64] Q. Ngo‐Metzger , M. P. Massagli , B. R. Clarridge , et al., “Linguistic and Cultural Barriers to Care,” Journal of General Internal Medicine 18, no. 1 (2003): 44–52. 12534763 10.1046/j.1525-1497.2003.20205.x PMC1494812

[65] M. Kwan , Z. Jeemi , R. Norman , and J. A. R. Dantas , “Professional Interpreter Services and the Impact on Hospital Care Outcomes: An Integrative Review of Literature,” International Journal of Environmental Research and Public Health 20, no. 6 (2023): 5165. 36982073 10.3390/ijerph20065165 PMC10048935

[66] L. Nadeau and T. Measham , “Immigrants and Mental Health Services: Increasing Collaboration With Other Service Providers,” Canadian Child and Adolescent Psychiatry Review 14, no. 3 (2005): 73–76. PMC2542908 19030510

[67] R. Vanstone and Victorian Foundation for Survivors of Torture and Forum of Australian Services for Survivors of Torture and Trauma , “Promoting Refugee Health: A Guide for Doctors, Nurses and Other Health Care Providers Caring for People From Refugee Backgrounds/[Writers Rachel Vanstone … [et al.]] Foundation House, Victorian Foundation for Survivors of Torture [Brunswick, Vic.],” (2012).

[68] Department of Health , Eligibility and Priority Access for Public Dental Services Policy (State Government of Victoria, 2014).

[69] S. Priebe , S. Sandhu , S. Dias , et al., “Good Practice in Health Care for Migrants: Views and Experiences of Care Professionals in 16 European Countries,” BMC Public Health 11 (2011): 187. 21439059 10.1186/1471-2458-11-187 PMC3071322

[70] R. B. Khatri and Y. Assefa , “Access to Health Services Among Culturally and Linguistically Diverse Populations in the Australian Universal Health Care System: Issues and Challenges,” BMC Public Health 22, no. 1 (2022): 880. 35505307 10.1186/s12889-022-13256-z PMC9063872

[71] A. Freiberg , A. Wienke , L. Bauer , A. Niedermaier , and A. Führer , “Dental Care for Asylum‐Seekers in Germany: A Retrospective Hospital‐Based Study,” International Journal of Environmental Research and Public Health 17, no. 8 (2020): 2672. 32295091 10.3390/ijerph17082672 PMC7215588

[72] Q. Nguyen , J. Flora , P. Basaviah , et al., “Interpreter and Limited‐English Proficiency Patient Training Helps Develop Medical and Physician Assistant Students' Cross‐Cultural Communication Skills,” BMC Medical Education 24, no. 1 (2024): 185. 38395858 10.1186/s12909-024-05173-z PMC10893691

