# Supplementary material for: A Systematic Review of the Facilitators and Challenges as Perceived by Dental Service Providers in the Provision of Oral Healthcare for Culturally and Linguistically Diverse Populations
Source: Community Dent Oral Epidemiol. 2025 Sep 3;54(1):7–21. doi: 10.1111/cdoe.70021 (PMC12808864; doi:10.1111/cdoe.70021)
Supplement: Supplementary file 1 — Table S1: Individual study quality assessments. Table S2: Themes and subthemes identified from the data analysis with example quotes. Table S3: Search strategies from various data sources. Table S4: Table of excluded studies. [file CDOE-54-7-s001.docx]

**Supplementary Information**

**Table S1: Individual study quality assessments**

|  | **Widstrom (1985)** | **Williams**  **et al (1995)** | **Goldsmith et al**  **(2005)** | **Zhang W**  **(2008)** | **Aljafari**  **et al (2015)** | **Due**  **et al (2020)** | **Van Midde et al (2020)** | **Imafuku**  **et al (2022)** | **Paajanen et al (2022)** | **Paisi et al (2022)** | **Spinler et al (2022)** | **Capozzi et al (2018)** | **Charbonneau et al**  **(2014)** |
| --- | --- | --- | --- | --- | --- | --- | --- | --- | --- | --- | --- | --- | --- |
| **S1** | Yes | Yes | Yes | Yes | Yes | Yes | Yes | Yes | Yes | Yes | Yes | Yes | Yes |
| **S2** | Yes | Yes | Yes | Yes | Yes | Yes | Yes | Yes | Yes | Yes | Yes | Yes | Yes |
| **1.1.** | **--** | **--** | **--** | Yes | Yes | Yes | Yes | Yes | Yes | Yes | Yes | Yes | Yes |
| **1.2.** | **--** | **--** | **--** | Yes | Yes | Yes | Yes | Yes | Yes | Yes | Yes | Yes | Yes |
| **1.3.** | **--** | **--** | **--** | No | Yes | Yes | Yes | Yes | Yes | Yes | Yes | Yes | Yes |
| **1.4.** | **--** | **--** | **--** | No | Yes | Yes | Yes | Yes | Yes | Yes | Yes | Yes | Yes |
| **1.5.** | **--** | **--** | **--** | Yes | Yes | Yes | Yes | Yes | Yes | Yes | Yes | Yes | Yes |
| **4.1.** | Yes | Yes | No | **--** | **--** | **--** | **--** | **--** | **--** | **--** | **--** | **--** | **--** |
| **4.2.** | Yes | Yes | Yes | **--** | **--** | **--** | **--** | **--** | **--** | **--** | **--** | **--** | **--** |
| **4.3.** | Yes | Yes | Yes | **--** | **--** | **--** | **--** | **--** | **--** | **--** | **--** | **--** | **--** |
| **4.4.** | Yes | No | No | **--** | **--** | **--** | **--** | **--** | **--** | **--** | **--** | **--** | **--** |
| **4.5.** | Yes | Yes | Yes | **--** | **--** | **--** | **--** | **--** | **--** | **--** | **--** | **--** | **--** |
|  | 1 | 0.8 | 0.6 | 0.6 | 1 | 1 | 1 | 1 | 1 | 1 | 1 | 1 | 1 |

***Screening Questions: S1:*** *Are there clear research questions? S2: Do the collected data allow to address the research questions?*

***Qualitative:*** *1.1. Is the qualitative approach appropriate to answer the research question? 1.2. Are the qualitative data collection methods adequate to address the research question? 1.3. Are the findings adequately derived from the data? 1.4. Is the interpretation of results sufficiently substantiated by data? 1.5. Is there coherence between qualitative data sources, collection, analysis, and interpretation?*

***Quantitative: 4.1.*** *Is the sampling strategy relevant to address the research question? 4.2. Is the sample representative of the target population? 4.3. Are the measurements appropriate? 4.4. Is the risk of nonresponse bias low? 4.5. Is the statistical analysis appropriate to answer the research question?*

**Table S2: Themes and subthemes identified from the data analysis with example quotes**

| **Theme** | **Sub-theme** | **Challenges/**  **Facilitators** | **Example Quote** |
| --- | --- | --- | --- |
| **Cultural factors** | Oral Health Literacy of CALD patients | Challenges | *“lack of knowledge of dental health is very common among them, and they do not see the importance of oral prevention.* (Zhang W. 2008) [43]  *“lack of oral health knowledge, unconsciousness of both the meaning and aims of oral health prevention.” (*Spinler et al., 2022) [47] |
|  | Awareness and understanding of available dental services | Challenges | “*a lack of patient awareness about ‘where to go for services’ and ‘who to contact*.” (Paisi et al., 2022) [39] |
|  | Cultural beliefs related to accessing dental services | Challenges | *“for example, the migrants do not believe that bacteria are the cause of caries but insist that air inside their bodies is the cause; moreover, some have religious beliefs about not filling or extracting teenagers’ teeth.”* (Zhang W. 2008) [43]  *“a lot of [refugees and asylum seekers] don't come back for regular sort of checks afterwards because that's not within their frame of reference, that's not what they're used to.”* (Due et al., 2020) [41]  *“male and female roles in other cultures may be different and might be hard to get through.”* (Capozzi et al., 2018) [48] |
|  | Beliefs and attitudes towards oral hygiene practices | Challenges | *“Oral health and oral hygiene are not a priority. The perceived significance of oral hygiene measures, the regular dental care of persons with a migration background is not comparable with the German population.”* (Spinler et al., 2022) [47] |
| **Language/ communication factors** | Establishing rapport | Challenges | *“Establishing rapport with migrant parents is challenging sometimes, and this was seen as a hindrance to the delivery of oral health care.”* (Aljafari et al., 2015) [38]  *“communication was much harder if they did not share a* *common language, It is more tiring and said that it took more energy and effort.”* (Paajanen et al., 2022) [46] |
|  | Past medical/ dental history | Challenges | “*. . . and they (persons with different cultural background) communicate their ailment different than, I would say, the German average citizen is doing it. Rather reserved or exuberant, depending on the entity staying in the background. This leads to the situation that the dentist sometimes does not know all relevant medical information.”* (Spinler et al., 2022) [47] |
|  | Means of communication | Challenges | *“least effective were the use of hand gestures or dictionaries.”* (Goldsmith et al., 2005) [40] |
|  |  | Facilitators | *“If you don’t know how to explain [something that comes up in the process of care] you can pull up the model [or diagrams] and use them.”* (Capozzi et al., 2018) [48] |
|  | Conveying treatment information | Challenges | “when foreign patients have a toothache, they want to pull it out right away because they couldn’t bear the pain, but, as a dentist, I see that some teeth can be treated without extraction. In such cases, it is very difficult to explain and persuade the patient to have dental treatment. Umm, if the patient was Japanese, it would be much easier to convey my opinion. As a dentist, I cannot extract teeth simply because of a toothache.” (Imafuku et al., 2022) [45] |
|  |  | Facilitators | “we have dental practice management software, which includes visual treatment planning with explanation videos. Using this software, various treatment procedures, such as root canals, and types of fillings can be visually explained. If I really want the patients to understand what I explain, I use this software. … I think it works to some extent.” (Imafuku et al., 2022) [45] |
|  | Compromised Informed consent | Challenges | *“I* *feel the patient would ask more questions if they could without going through an interpreter. Because of the language barrier, I believe the patient accepts the treatment without questioning.”* (Goldsmith et al., 2005) [40] |
|  | Interpreter-mediated communication | Challenges | “I’m not sure to what extent the interpreter, like the patient’s family and friends, can understand what I said. Moreover, when the interpreter translates my words into the patient’s language, I totally don’t know the accuracy of the translation. In the end, I’m worried whether the patient understood what I’m saying through an interpreter.” (Imafuku et al., 2022) [45] |
|  |  | Facilitators | *“Professional interpreters have been successful and have clear explanations, providing better-informed consent.”* (Goldsmith et al., 2005) [40]  *“I would like to have an interpreter in dental examination appointments, to reach a conclusion about (the patient’s) expectations and hopes and, on the other hand, tell what I think I can do, and I would like to do as well as what is possible to do.”* (Paajanen et al., 2022) [46] |
| **Psychosocial factors** | Selfless commitment/ compassionate toward migrant patients | Facilitators | *“I like the idea of doing something useful for people who can't afford to pay for oral care, especially when it concerns basic treatment.”* (van Midde et al., 2021) [44]  *“Don’t get frustrated, it’s not going to be easy” and “Keep an open mind” and, “[Remember to] … not get overwhelmed.”* (Capozzi et al., 2018) [48] |
|  | Help-seeking attitude | Challenges | *“the biggest block has always been communication for these people. So even when they have arrived here, knowing we have a full range of facilities, there is a little bit of anxiety in, in going out and seeking help etc.”* (Aljafari et al., 2015) [38] |
|  |  | Facilitators | *“many dentists responded* *that patients should seek out dentists who speak their own language and they should take more initiative for their own needs and secure their own interpreters.”* (Goldsmith et al., 2005) [40] |
|  | Lack of gratitude/ Frustration & Fear | Challenges | *“When you reserve your time to help someone, you also expect that person to be grateful instead of showing an unhappy face.”* (van Midde et al., 2021) [44]  *“gaining consent and having limited access to ‘interpretation services,’ or ‘information in different languages’ that may lead to a degree of apprehension, fear and frustration among dentists.”* (Paisi et al., 2022) [40] |
|  | Treatment Decision-making/ Ethical dilemmas | Challenges | “The foreign patients might want to say something to me. After the dental treatment, I really feel like it was unfinished business. If the patient is Japanese, I can convey what I want to say. … Actually, I want to treat them as I do Japanese patients, but I have no choice but to focus on completing their treatment quickly. That’s why I’m unsatisfied with my practice with the foreign patients.” (Imafuku et al., 2022) [45] |
|  | Expectations vs Reality | Challenges | “One of the biggest challenges is explaining health insurance system in Japan to foreign patients. Particularly, people from Brazil dislike silver fillings. … So, if they wish to have white fillings, it would be at their own expense. In this situation, it is difficult to explain why it cannot be offered within the insurance application range.” (Imafuku et al., 2022) [45] |
|  | Previous dental experiences | Challenges | *“she* *went to the dentist…and had the wrong tooth pulled out!’ affected trust and confidence in the profession, with people becoming reluctant to seek dental care: ‘she had no faith in going back.”* (Paisi et al., 2022) [39] |
|  | Resettlement/ traumatic past | Challenges | *“oral health may ‘not [be] a priority’ for asylum seekers and refugees until they ‘are finally safe,’ both in terms of their living situation and asylum seeker case progression.”* (Paisi et al., 2022) [39] |
| **Structural and system determinants** | Dentist training | Facilitators | *“We need specific lectures to give us insight and views on specific cultures.”* (Capozzi et al., 2018) [48] |
|  | Burden/ loss to the clinics | Challenges | *“Having staff take time away from their regular duties to either bring in an interpreter or to serve as an interpreter themselves was also viewed as an economic loss to the practice.”* (Goldsmith et al., 2005) [40] |
|  | Oral Health Education Information | Challenges | *“there is a limited ‘availability of information in different languages.’ Asylum seekers and refugees’ lack of awareness that they needed to seek routine care was also reported, ‘there is not enough education on people, to say you are required to have your teeth checked in this length of time.”* (Paisi et al., 2022) [39] |
|  |  | Facilitators | *“providing oral care education information ‘in different languages,’ supported by culturally diverse, ‘appropriate and sensitive photographs, images and graphics’ was considered essential.”* (Paisi et al., 2022) [39] |
|  | System support | Challenges | *“I don’t think the NHS does a particularly good job of communicating out through local authorities and with organisations that support vulnerable groups. So, often organisations themselves don’t know how to access dental services or how to signpost individuals to advise on oral health.”* (Paisi et al., 2022) [39] |
|  |  | Facilitators | *“a positive incentive payment scheme might be offered to the dentists in recognition of the population’s needs.”* (Williams et al., 1995) [37] |
|  | Access to the services | Challenges | *“Challenges in getting urgent appointments ‘for people with infections and with swollen faces’ were also raised.”* (Paisi et al., 2022) [39] |
|  |  | Facilitators | *“improving awareness of service location and transportation could improve service access.”*(Paisi et al., 2022) [39] |
|  | Key Partnerships & Meaningful Relationships | Facilitators | *“Pairing patients with dentists who spoke the same language could be vital to avoid any cultural barriers.”* (Paisi et al., 2022) [39] |
| **Affordability** | Cost | Challenges | *“The cost of dental care was viewed as the greatest barrier for the migrants.”* (Zhang W. 2008) [43]  “*out of pocket payment for treatments would reinforce the avoidance for dental check-ups, or they delay a necessary visit of the dentist until it is unavoidable.”* (Spinler et al., 2022) [47] |
|  | Useful resources | Challenges | *“dentists acknowledged that migrants were unable to have the luxury of choosing what to eat’ when relying on ‘helping organisations’ that often provide ‘food rations that are high-sugar based’ or forced to choose ‘foods that have long preserving lives because they can’t afford’ fresh fruit and vegetables.”* (Paisi et al., 2022) [39]  *“‘I had the mother of a little girl who had seven teeth pulled out because she – they weren’t looking after her teeth properly. And it was again this thing about trying to manage on a very small budget and buying food that wasn’t really helpful.”* (Paisi et al., 2022) [39] |

**Table S3: Search strategies from various data sources**

**Search strategy from EMBASE:**

| 1 | CALD.mp. | 736 |
| --- | --- | --- |
| 2 | cultural* and linguistic*.mp. [mp=title, abstract, heading word, drug trade name, original title, device manufacturer, drug manufacturer, device trade name, keyword heading word, floating subheading word, candidate term word] | 3531 |
| 3 | non-English speak*.mp. [mp=title, abstract, heading word, drug trade name, original title, device manufacturer, drug manufacturer, device trade name, keyword heading word, floating subheading word, candidate term word] | 2147 |
| 4 | foreign-born.mp. [mp=title, abstract, heading word, drug trade name, original title, device manufacturer, drug manufacturer, device trade name, keyword heading word, floating subheading word, candidate term word] | 4470 |
| 5 | refugee*.mp. [mp=title, abstract, heading word, drug trade name, original title, device manufacturer, drug manufacturer, device trade name, keyword heading word, floating subheading word, candidate term word] | 21051 |
| 6 | asylum seeker*.mp. [mp=title, abstract, heading word, drug trade name, original title, device manufacturer, drug manufacturer, device trade name, keyword heading word, floating subheading word, candidate term word] | 2774 |
| 7 | migrant*.mp. [mp=title, abstract, heading word, drug trade name, original title, device manufacturer, drug manufacturer, device trade name, keyword heading word, floating subheading word, candidate term word] | 32933 |
| 8 | Emigrants.mp. | 17853 |
| 9 | Immigrants.mp. [mp=title, abstract, heading word, drug trade name, original title, device manufacturer, drug manufacturer, device trade name, keyword heading word, floating subheading word, candidate term word] | 31052 |
| 10 | foreign-background.mp. [mp=title, abstract, heading word, drug trade name, original title, device manufacturer, drug manufacturer, device trade name, keyword heading word, floating subheading word, candidate term word] | 93 |
| 11 | 1 or 2 or 3 or 4 or 5 or 6 or 7 or 8 or 9 or 10 | 83693 |
| 12 | dentist*.mp. [mp=title, abstract, heading word, drug trade name, original title, device manufacturer, drug manufacturer, device trade name, keyword heading word, floating subheading word, candidate term word] | 151972 |
| 13 | dental service provider*.mp. [mp=title, abstract, heading word, drug trade name, original title, device manufacturer, drug manufacturer, device trade name, keyword heading word, floating subheading word, candidate term word] | 34 |
| 14 | dental expert*.mp. [mp=title, abstract, heading word, drug trade name, original title, device manufacturer, drug manufacturer, device trade name, keyword heading word, floating subheading word, candidate term word] | 140 |
| 15 | dental practi*.mp. [mp=title, abstract, heading word, drug trade name, original title, device manufacturer, drug manufacturer, device trade name, keyword heading word, floating subheading word, candidate term word] | 16657 |
| 16 | oral health practitioner*.mp. [mp=title, abstract, heading word, drug trade name, original title, device manufacturer, drug manufacturer, device trade name, keyword heading word, floating subheading word, candidate term word] | 88 |
| 17 | stakeholders.mp. [mp=title, abstract, heading word, drug trade name, original title, device manufacturer, drug manufacturer, device trade name, keyword heading word, floating subheading word, candidate term word] | 69955 |
| 18 | 12 or 13 or 14 or 15 or 16 or 17 | 229280 |
| 19 | 11 and 18 | 1312 |
| 20 | experience*.mp. [mp=title, abstract, heading word, drug trade name, original title, device manufacturer, drug manufacturer, device trade name, keyword heading word, floating subheading word, candidate term word] | 1544361 |
| 21 | challenge*.mp. [mp=title, abstract, heading word, drug trade name, original title, device manufacturer, drug manufacturer, device trade name, keyword heading word, floating subheading word, candidate term word] | 1143581 |
| 22 | barrier*.mp. [mp=title, abstract, heading word, drug trade name, original title, device manufacturer, drug manufacturer, device trade name, keyword heading word, floating subheading word, candidate term word] | 495111 |
| 23 | difficult*.mp. [mp=title, abstract, heading word, drug trade name, original title, device manufacturer, drug manufacturer, device trade name, keyword heading word, floating subheading word, candidate term word] | 859822 |
| 24 | perspective*.mp. [mp=title, abstract, heading word, drug trade name, original title, device manufacturer, drug manufacturer, device trade name, keyword heading word, floating subheading word, candidate term word] | 540080 |
| 25 | perception*.mp. [mp=title, abstract, heading word, drug trade name, original title, device manufacturer, drug manufacturer, device trade name, keyword heading word, floating subheading word, candidate term word] | 580574 |
| 26 | facilitator*.mp. [mp=title, abstract, heading word, drug trade name, original title, device manufacturer, drug manufacturer, device trade name, keyword heading word, floating subheading word, candidate term word] | 48143 |
| 27 | 20 or 21 or 22 or 23 or 24 or 25 or 26 | 4452564 |
| 28 | 19 and 27 | 850 |
| 29 | mixed-method*.mp. [mp=title, abstract, heading word, drug trade name, original title, device manufacturer, drug manufacturer, device trade name, keyword heading word, floating subheading word, candidate term word] | 56383 |
| 30 | qualitative study.mp. [mp=title, abstract, heading word, drug trade name, original title, device manufacturer, drug manufacturer, device trade name, keyword heading word, floating subheading word, candidate term word] | 77395 |
| 31 | quantitative study.mp. [mp=title, abstract, heading word, drug trade name, original title, device manufacturer, drug manufacturer, device trade name, keyword heading word, floating subheading word, candidate term word] | 10867 |
| 32 | 29 or 30 or 31 | 141775 |
| 33 | 28 and 32 | 203 |

**Search strategy from CINAHL:**

| **#** | **Query** | **Results** |
| --- | --- | --- |
| S1 | TX "CALD" OR TX ( "cultural* and linguistic*" ) OR TX "non-English speak*" OR TX "foreign-born" OR TX "foreign-background" OR TX "asylum seeker*" OR TX "refugee*" OR TX "migrant*" OR TX ( "Emigrants and Immigrant*" ) | 88959 |
| S2 | TX "dentist*" OR TX "dental service provider*" OR TX "dental expert*" OR TX "dental practi*" OR TX "oral health practi*" OR TX "stakeholders" | 338879 |
| S3 | TX "experience*" OR TX "challenge*" OR TX "barrier*" OR TX "difficult*" OR TX "perspective*" OR TX "perception*" | 2666719 |
| S4 | TX "mixed-method*" OR TX "qualitative study" OR TX "qualitative research" OR TX "qualitative survey" OR TX "quantitative study" | 295820 |
| S5 | S1 AND S2 AND S3 AND S4 | 5233 |

**Search Strategy from SCOPUS:**

| ( ( TITLE-ABS-KEY ( ( "mixed-method*" ) ) ) OR ( TITLE-ABS-KEY ( ( "qualitative study" ) ) ) OR ( TITLE-ABS-KEY ( ( "quantitative study" ) ) ) ) AND ( ( ( ( TITLE-ABS-KEY ( ( "foreign-born" ) ) ) OR ( TITLE-ABS-KEY ( ( "foreign-background" ) ) ) OR ( TITLE-ABS-KEY ( ( "asylum seeker*" ) ) ) OR ( TITLE-ABS-KEY ( ( "migrant*" ) ) ) OR ( TITLE-ABS-KEY ( "CALD" ) ) OR ( TITLE-ABS-KEY ( ( "non-English speak*" ) ) ) OR ( TITLE-ABS-KEY ( ( "cultural* and linguistic*" ) ) ) OR ( TITLE-ABS-KEY ( ( "refugee*" ) ) ) OR ( TITLE-ABS-KEY ( ( "Emigrants and immigrant" ) ) ) ) AND ( ( TITLE-ABS-KEY ( ( "dentist*" ) ) ) OR ( TITLE-ABS-KEY ( ( "dental service provider*" ) ) ) OR ( TITLE-ABS-KEY ( ( "dental expert*" ) ) ) OR ( TITLE-ABS-KEY ( ( "dental practi*" ) ) ) OR ( TITLE-ABS-KEY ( ( "oral health practi*" ) ) ) OR ( TITLE-ABS-KEY ( ( "stakeholders" ) ) ) ) ) AND ( ( TITLE-ABS-KEY ( ( "experience*" ) ) ) OR ( TITLE-ABS-KEY ( ( "difficult*" ) ) ) OR ( TITLE-ABS-KEY ( ( "perspective*" ) ) ) OR ( TITLE-ABS-KEY ( ( "perception*" ) ) ) OR ( TITLE-ABS-KEY ( ( "challenge*" ) ) ) OR ( TITLE-ABS-KEY ( ( "barrier*" ) ) ) OR ( TITLE-ABS-KEY ( ( "facilitator*" ) ) ) ) ) | 301 |
| --- | --- |

**Search Strategy from WEB OF SCIENCE:**

|  |  |  |
| --- | --- | --- |
| 1 | **"CALD"** (All Fields) or **"cultural* and linguistic*"** (All Fields) or **"non-English speak*"** (All Fields) or **"foreign-born"** (All Fields) or **"foreign-background"** (All Fields) or **"asylum seeker*"** (All Fields) or **"refugee*"** (All Fields) or **"migrant*"** (All Fields) or **"Emigrants and Immigrant"** (All Fields) | 1667 |
| 2 | **"dentist*"** (All Fields) or **"dental service provider*"** (All Fields) or **"dental expert*"** (All Fields) or **"dental practi*"** (All Fields) or **"oral health practi*"** (All Fields) or **"stakeholders"** (All Fields) | 8589 |
| 3 | **"experience*"** (All Fields) or **"challenge*"** (All Fields) or **"barrier*"** (All Fields) or **"difficult*"** (All Fields) or **"perspective*"** (All Fields) or **"perception*"** (All Fields) or **"facilitator*"** (All Fields) | 191366 |
| 4 | **"mixed-method*"** (All Fields) or **"qualitative study"** (All Fields) or **"quantitative study"** (All Fields) | 3641 |
| 5 | **#1 AND #2 AND #3 AND #4** | 4 |

**Search Strategy from PROQUEST:**

|  |  |
| --- | --- |
| noft("CALD") OR noft("cultural* and linguistic*") OR noft("non-English speak*" ) OR noft("foreign-born" ) OR noft("foreign background") OR noft("asylum seeker*") OR noft("refugee*") OR noft("migrant*") AND noft("dentist*" ) OR noft("dental service provider*") OR noft("dental expert*" ) OR noft("dental practi*" ) OR noft("oral health practi*" ) OR noft("stakeholders") AND noft("experience*" ) OR noft("challenge*") OR noft("barrier*") OR noft("difficult*") OR noft("perspective*") OR noft("perception*") AND noft("facilitator*") AND noft("mixed-method*" ) OR noft("qualitative study" ) OR noft("quantitative study" ) | 675 |

**Table S4: Table of excluded studies**

| **S.no** | **Study details** | **Reason for Exclusion** |
| --- | --- | --- |
|  | Al-Rousan T, Schwabkey Z, Jirmanus L, Nelson BD. Health needs and priorities of Syrian refugees in camps and urban settings in Jordan: perspectives of refugees and health care providers. Eastern Mediterranean Health Journal. 2018;24(3):243-53. | Perspectives of general healthcare providers & stakeholders |
|  | Aldukhail S, Shukla A, Khadra MT, Ziad Al H, Jordan S, Cadet TJ, Alqaderi H. Oral and emotional health experience of refugees’ in the state of Massachusetts - A mixed methods approach. PLoS One. 2023;18(3). | Oral Health experiences of refugees/migrants |
|  | Arize I, Ogbuabor D, Mbachu C, Etiaba E, Uzochukwu B, Onwujekwe O. Stakeholders' Perspectives on the Unmet Needs and Health Priorities of the Urban Poor in South-East Nigeria. Community Health Equity Research & Policy. 2023;43(4):389-98. | Perspectives of general healthcare providers & stakeholders |
|  | Arora A, Rana K, Manohar N, Li L, Bhole S, Chimoriya R. Perceptions and Practices of Oral Health Care Professionals in Preventing and Managing Childhood Obesity. Nutrients. 2022;14(9):1809-. | Not on CALD population |
|  | Badri P, Amin M, Wolfe R, Farmer A. Psychosocial Determinants of Adherence to Preventive Dental Attendance for Preschool Children Among Filipino Immigrants in Edmonton, Alberta. Journal of Immigrant & Minority Health. 2018;20(3):658-67. | Oral Health experiences of refugees/migrants |
|  | Braun PA, Budzyn SE, Chavez C, Barnard JG. Integrating Dental Hygienists into Medical Care Teams: Practitioner and patient perspectives. Journal of Dental Hygiene. 2021;95(3):6-17. | Not on CALD population |
|  | Browne‐Yung K, O'Neil D, Walker R, Corlis M, Smyth A, Putsey P, et al. Perspectives of professionals on the safety and accessibility of aged care for Care Leavers and Forgotten Australians. Australasian Journal on Ageing. 2022;41(1):42-9. | Perspectives of general healthcare providers & stakeholders |
|  | Charbonneau CJ, Neufeld MJ, Craig BJ, Donnelly LR. Increasing cultural competence in the dental hygiene profession. Canadian Journal of Dental Hygiene. 2009;43(6):297-305. | Review |
|  | Ferguson C, George A, Villarosa AR, Kong AC, Bhole S, Ajwani S. Exploring nursing and allied health perspectives of quality oral care after stroke: A qualitative study. European Journal of Cardiovascular Nursing. 2020;19(6):505-12. | Not on CALD population |
|  | Filmer TR, Ray RA, Glass BD. How can we help you? A qualitative study of the provision of care to culturally and linguistically diverse clients in community pharmacy. International Journal of Pharmacy Practice. 2023;31(6):601-7. | Perspectives of pharmacists/nurses |
|  | Finnegan D, Rainchuso L, Jenkins S, Kierce E, Rothman A. Immigrant Caregivers of Young Children: Oral Health Beliefs, Attitudes, and Early Childhood Caries Knowledge. Journal of Community Health. 2016;41(2):250-7. | Oral Health experiences of refugees/migrants |
|  | Finney Lamb CE, Whelan AK, Michaels C. Refugees and oral health: Lessons learned from stories of Hazara refugees. Australian Health Review. 2009;33(4):618-27. | Oral Health experiences of refugees/migrants |
|  | Gaya-Sancho B, Vanceulebroeck V, Komurcu N, Kalkan I, Casa-Nova A, Tambo-Lizalde E, et al. Perception and experience of transcultural care of stakeholders and health service users with a migrant background: A qualitative study. International Journal of Environmental Research and Public Health. 2021;18(19):10503. | Perspectives of general healthcare providers & stakeholders |
|  | Ghiabi E, Matthews D, Brillant M. The Oral Health Status of Recent Immigrants and Refugees in Nova Scotia, Canada. Journal of Immigrant & Minority Health. 2014;16(1):95-101. | Oral Health experiences of refugees/migrants |
|  | Hahn K, Steinhauser J, Goetz K. Equity in Health Care: A Qualitative Study with Refugees, Health Care Professionals, and Administrators in One Region in Germany. BioMed Research International. 2020;2020:4647389. | Perspectives of general healthcare providers & stakeholders |
|  | Hémono R, Relyea B, Scott J, Khaddaj S, Douka A, Wringe A. "The needs have clearly evolved as time has gone on.": A qualitative study to explore stakeholders' perspectives on the health needs of Syrian refugees in Greece following the 2016 European Union-Turkey agreement. Conflict and Health. 2018;12:9. | Perspectives of general healthcare providers & stakeholders |
|  | Kaihlanen A-M, Hietapakka L, Heponiemi T. Increasing cultural awareness: qualitative study of nurses' perceptions about cultural competence training. BMC Nursing. 2019;18(1):N.PAG-N.PAG. | Perspectives of pharmacists/nurses |
|  | Kamau S, Oikarainen A, Juntunen MM, Koskenranta M, Kuivila H, Tomietto M, Mikkonen K. Nurse educators' views of integrating culturally and linguistically diverse future registered nurses into healthcare settings: A qualitative descriptive study. Journal of Advanced Nursing (John Wiley & Sons, Inc). 2023;79(9):3412-25. | Perspectives of pharmacists/nurses |
|  | Kaur N, Kandelman D, Potvin L. Effectiveness of "Safeguard Your Smile," an oral health literacy intervention, on oral hygiene self-care behaviour among Punjabi immigrants: A randomized controlled trial. Canadian Journal of Dental Hygiene. 2019;53(1):23-32. | Effectivness of OH Intervention |
|  | Khaw SML, Homer CSE, Dearnley RE, O'Rourke K, Akter S, Bohren MA. A qualitative study on community-based doulas' roles in providing culturally-responsive care to migrant women in Australia. Women and Birth. 2023;36(5):e527-e35. | Perspectives of general healthcare providers & stakeholders |
|  | Kidane YS, Ziegler S, Keck V, Benson-Martin J, Jahn A, Gebresilassie T, Beiersmann C. Eritrean refugees' and asylum-seekers' attitude towards and access to oral healthcare in heidelberg, germany: A qualitative study. International Journal of Environmental Research and Public Health. 2021;18(21):11559. | Oral Health experiences of refugees/migrants |
|  | Kim Yen TN, Smallidge DL, Boyd LD, Rainchuso L. Vietnamese Oral Health Beliefs and Practices: Impact on the Utilization of Western Preventive Oral Health Care. Journal of Dental Hygiene. 2017;91(1):49-56. | Oral Health experiences of refugees/migrants |
|  | Lamb CEF, Whelan AK, Michaels C. Refugees and oral health: lessons learned from stories of Hazara refugees. Australian Health Review. 2009;33(4):618-27. | Oral Health experiences of refugees/migrants |
|  | Lamb CF, Phelan C. Cultural observations on Vietnamese children's oral health practices and use of the child oral health services in Central Sydney: A qualitative study. Australian Journal of Primary Health. 2008;14(1):75-81. | Oral Health experiences of refugees/migrants |
|  | Lewney J, Holmes RD, Rankin J, Exley C. Health visitors' views on promoting oral health and supporting clients with dental health problems: a qualitative study. Journal of Public Health. 2019;41(1):e103-e8. | Not on CALD population |
|  | Li Q, Du M, Knight JC, Yi Y, Wang Q, Wang PP, Zhu Y. Dental Insurance Coverage, Dentist Visiting, and Oral Health Status among Asian Immigrant Women of Childbearing Age in Canada: A Comparative Study. Healthcare (2227-9032). 2023;11(19):2666. | Oral Health experiences of refugees/migrants |
|  | Lieneck C, Connelly E, Ireland D, Jefferson A, Jones J, Breidel N. Facilitators and Barriers to Oral Healthcare for Women and Children with Low Socioeconomic Status in the United States: A Narrative Review. Healthcare (2227-9032). 2023;11(16):2248. | Not on CALD population |
|  | Macdonald ME, Keboa MT, Nurelhuda NM, Lawrence HP, Carnevale F, McNally M, et al. The oral health of refugees and Asylum seekers in Canada: A mixed methods study protocol. International Journal of Environmental Research and Public Health. 2019;16(4):542. | Oral Health experiences of refugees/migrants |
|  | Mariño R, Minichiello V, MacEntee MI. Understanding oral health beliefs and practices among Cantonese-speaking older Australians. Australasian Journal on Ageing. 2010;29(1):21-6. | Oral Health experiences of refugees/migrants |
|  | Marquès-Pellejà G, Roqueta-Vall-llosera M, Cámara-Liebana D, Mantas-Jiménez S, Gelabert-Vilella S, Baltasar-Bagué A, et al. Assessing the Student Nurses' Knowledge of Oral Health Care. Nursing Reports. 2023;13(3):1126-37. | Perspectives of pharmacists/nurses |
|  | McCann J, Lau WM, Husband A, Todd A, Sile L, Doll AK, et al. 'Creating a culturally competent pharmacy profession': A qualitative exploration of pharmacy staff perspectives of cultural competence and its training in community pharmacy settings. Health Expectations. 2023;26(5):1941-53. | Perspectives of general healthcare providers & stakeholders |
|  | Mofidi M, Zeldin LP, Rozier RG. Oral health of Early Head Start children: a qualitative study of staff, parents, and pregnant women. American Journal of Public Health. 2009;99(2):245-51. | Oral Health experiences of refugees/migrants |
|  | Montayre J, De‐Arth J, Shrestha‐Ranjit J, Neville S, Holroyd E. Challenges and adjustments in maintaining health and well‐being of older Asian immigrants in New Zealand: An integrative review. Australasian Journal on Ageing. 2019;38(3):154-72. | review |
|  | Moreau A-M, Hennous F, Dabbagh B, Ferraz dos Santos B. Oral Health Status of Refugee Children in Montreal. Journal of Immigrant & Minority Health. 2019;21(4):693-8. | Oral Health experiences of refugees/migrants |
|  | Murray K, Khawaja NG, Schweitzer RD, Baldwin L, Bentley S. Provider perspectives on services for people seeking asylum in Australia: best practices and challenges. Aust Psychol. 2021;56(4):289-98. | Perspectives of general healthcare providers & stakeholders |
|  | Oda K, Boyd M, Parsons J, Smith M. Integrating oral care into nursing practice in care homes. Nursing Older People. 2022;34(4):27-34. | Review |
|  | Ogata A, Naiki M, Onzima A, Saito Y. Stakeholders’ perceived achievements and challenges after the safe motherhood project in Northern Uganda. African Journal of Reproductive Health. 2022;26(11):23-31. | Perspectives of general healthcare providers & stakeholders |
|  | Olerud E, Hagman‐Gustavsson ML, Gabre P. Experience of dental care, knowledge and attitudes of older immigrants in Sweden—A qualitative study. International Journal of Dental Hygiene. 2018;16(2):e103-e11. | Oral Health experiences of refugees/migrants |
|  | Peiris-John R, Wong A, Sobrun-Maharaj A, Ameratunga S. Stakeholder views on factors influencing the wellbeing and health sector engagement of young Asian New Zealanders. Journal of Primary Health Care. 2016;8(1):35-43. | Not on CALD population |
|  | Peprah P, Lloyd J, Harris M. Health literacy and cultural responsiveness of primary health care systems and services in Australia: reflections from service providers, stakeholders, and people from refugee backgrounds. Bmc Public Health. 2023;23(1):18. | Perspectives of general healthcare providers & stakeholders |
|  | Petts RA, Lewis RK, Brooks K, McGill S, Lovelady T, Galvez M, Davis E. Examining Patient and Provider Experiences with Integrated Care at a Community Health Clinic. Journal of Behavioral Health Services & Research. 2022;49(1):32-49. | Not on CALD population |
|  | Pham K, Barker JC, Lazar AA, Walsh M. Oral Health Care of Vietnamese Adolescents: A Qualitative Study of Perceptions and Practices. Journal of Dental Hygiene. 2015;89(6):397-404. | Oral Health experiences of refugees/migrants |
|  | Pittala R, Jacob W. The need for inclusion of integrated teaching on refugee and asylum seeker health in undergraduate medical curriculum. Frontiers in Education. 2023;8:12. | Perspectives of general healthcare providers & stakeholders |
|  | Raddawi R. Intercultural (Mis-) communication in medical settings: Cultural difference or cultural incompetence? Intercultural Communication with Arabs: Studies in Educational, Professional and Societal Contexts2015. p. 179-95. | Perspectives of general healthcare providers & stakeholders |
|  | Reich SM, Ochoa W, Gaona A, Salcedo Y, Espino Bardales G, Newhart V, et al. Disparities in Caregivers' Experiences at the Dentist With Their Young Child. Academic Pediatrics. 2019;19(8):969-77. | Not on CALD population |
|  | Riggs E, Gibbs L, Kilpatrick N, Gussy M, van Gemert C, Ali S, Waters E. Breaking down the barriers: a qualitative study to understand child oral health in refugee and migrant communities in Australia. Ethnicity & Health. 2015;20(3):241-57. | Oral Health experiences of refugees/migrants |
|  | Rivera Y, Boyd LD, Libby L. Hispanic Seasonal Farmworker Caregivers' Beliefs and Perceptions of Early Childhood Caries. Journal of Dental Hygiene. 2020;94(5):14-21. | Oral Health experiences of refugees/migrants |
|  | Rogo EJ, Hodges KO, Evans JL. Patients' Perspectives About the Influence of Dental Hygienists' Social Intelligence on Self-Care. Journal of Dental Hygiene. 2022;96(6):24-33. | Not on CALD population |
|  | Sangaramoorthy T, Guevara E. Immigrant Health in Rural Maryland: A Qualitative Study of Major Barriers to Health Care Access. Journal of Immigrant & Minority Health. 2017;19(4):939-46. | Perspectives of general healthcare providers & stakeholders |
|  | Schiavo JH. Oral Health Literacy in the Dental Office: The Unrecognized Patient Risk Factor. Journal of Dental Hygiene. 2011;85(4):248-55. | Review |
|  | Schneider AA, Meira CGD, Galli FL, Mello ALSF, Pilati SFM. Oral health and health care in female sex workers: concomitant quantitative and qualitative approaches. Women & Health. 2021;61(9):880-8. | Not on CALD population |
|  | Shahawy S, Onwuzurike C, Premkumar A, Henricks AA, Simon MA. Perspectives of women of refugee background on healthcare needs in a major urban metropolitan community in the US: A qualitative needs assessment. Health & Social Care in the Community. 2022;30(6):e5637-e46. | Oral Health experiences of refugees/migrants |
|  | Trudnak Fowler T, Matthews G, Black C, Crosby Kowal H, Vodicka P, Edgerton E. Evaluation of a Comprehensive Oral Health Services Program in School-Based Health Centers. Maternal & Child Health Journal. 2018;22(7):998-1007. | Effectiveness of OH Intervention |
|  | Vamos C, Walsh M, Thompson E, Daley E, Detman L, DeBate R. Oral-Systemic Health During Pregnancy: Exploring Prenatal and Oral Health Providers' Information, Motivation and Behavioral Skills. Maternal & Child Health Journal. 2015;19(6):1263-75. | Not on CALD population |
|  | Balla SB, Tadakamadla J, Tadakamadla SK. 'The Letter Says I May or May Not Be Eligible… It Is a Big Doubt and Frustrating:' A Qualitative Study on Barriers and Facilitators to Children's Oral Healthcare From the Perspective of Karen Refugee Parents in Victoria. Health Expect. 2024 Dec;27(6):e70110. | Oral Health experiences of refugees/migrants |
|  | Liu ZE. Newcomers' perceptions of their experiences with oral health care in Canada and the United States. Can J Dent Hyg. 2024 Oct 1;58(3):196-206. PMID: 39513096; PMCID: PMC11539944. | Review |
|  | Van Dam L, Diab E, Johnson J. Canadian immigrants' oral health and oral health care providers' cultural competence capacity. Can J Dent Hyg. 2024 Feb 1;58(1):34-47. | Review |
|  | Muller R, Bilich L, Jones M. Impact of an Oral Health Education Program on the Oral Health Literacy of Refugees. J Immigr Minor Health. 2024 Aug;26(4):699-710. | Effectiveness of OH Intervention |
|  | Marcus K, Balasubramanian M, Short SD, Sohn W. Quantitative analysis on dental utilisation in culturally and linguistically diverse mothers. Aust J Prim Health. 2024 Feb;30(1):NULL. | Oral Health experiences of refugees/migrants |
